# The Identification and Characterization of Endopolygalacturonases in a South African Isolate of *Phytophthora cinnamomi*

**DOI:** 10.3390/microorganisms10051061

**Published:** 2022-05-20

**Authors:** Tsakani Magdeline Miyambo, Robert Backer, Juanita Engelbrecht, Fourie Joubert, Nicolaas Albertus van der Merwe, Noëlani van den Berg

**Affiliations:** 1 Department of Biochemistry, Genetics and Microbiology, University of Pretoria, Pretoria 0028, South Africa; tsakane.miyambo@fabi.up.ac.za (T.M.M.); robert.backer@fabi.up.ac.za (R.B.); juanita.engelbrecht@fabi.up.ac.za (J.E.); fourie.joubert@up.ac.za (F.J.); albe.vdmerwe@fabi.up.ac.za (N.A.v.d.M.); 2 Forestry and Agricultural Biotechnology Institute, University of Pretoria, Pretoria 0028, South Africa; 3 Centre for Bioinformatics and Computational Biology, University of Pretoria, Pretoria 0028, South Africa

**Keywords:** *Phytophthora cinnamomi*, endopolygalacturonases, bioinformatics, evolution, phylogenetics

## Abstract

*Phytophthora cinnamomi* is an economically important plant pathogen that has caused devastating losses to the avocado industry worldwide. To facilitate penetration and successful colonization of the host plant, pathogens have been reported to secrete polygalacturonases (PGs). Although a large PG gene family has been reported in *P. cinnamomi*, in-depth bioinformatics analyses and characterization of these genes is still lacking. In this study we used bioinformatics tools and molecular biology techniques to identify and characterize endopolygalacturonases in the genome of a South African *P. cinnamomi* isolate, GKB4. We identified 37 PGs, with 19 characteristics of full-length PGs. Although eight PcPGs were induced in planta during infection, only three showed significant up- and down-regulation when compared with in vitro mycelial growth, suggesting their possible roles in infection. The phylogenetic analysis of PcPGs showed both gain and loss of introns in the evolution of PGs in *P. cinnamomi*. Furthermore, 17 PGs were related to characterized PGs from oomycete species, providing insight on possible function. This study provides new data on endoPGs in *P. cinnamomi* and the evolution of introns in PcPG genes. We also provide a baseline for future functional characterization of PGs suspected to contribute to *P. cinnamomi* pathogenicity/virulence in avocado.

## 1. Introduction

*Phytophthora cinnamomi* Rands is an oomycete pathogen that causes disease in a number of plant species in agriculture, forestry and natural ecosystems worldwide [1]. This pathogen is the causal agent of Phytophthora root rot (PRR) in *Persea americana* Mill (avocado). Due to the wide host range of *P. cinnamomi*, especially among the woody ornamental plants, it remains a continuous threat to the avocado industry in countries where the disease is prevalent. The pathogen causes symptoms such as wilting, canker and dieback, which results in the eventual death of the host plant. Basic details of the infection strategy and defense response during the oomycete–plant interaction have been known for some time [2]. However, a careful understanding of the biology of the pathogen and important pathogenicity or virulence genes employed for successful infection is necessary.

A critical aspect of plant infection by many phytopathogens, such as *P. cinnamomi*, is penetration of the physical barrier of the plant cell wall [3]. Penetration is facilitated by the secretion of a variety of cell wall degrading enzymes (CWDEs). Some of the very first CWDEs to be secreted by the pathogen during infection are those that break down the pectin component of the plant cell wall [4]. Polygalacturonases (PGs), are one of the first pectic enzymes secreted by the pathogen during infection. These enzymes have been reported as virulence/pathogenicity factors [5,6,7,8,9], while others have contradicted this [10,11,12]. Therefore, the exact role of PGs in virulence is not fully understood and is likely to differ depending on the pathosystem being studied [13]. In the *P. cinnamomi*-avocado interaction, we expect few PGs to play a significant role in the early stage of pathogen penetration and colonization; meanwhile, more PGs are expected to be observed during the necrotrophic phase.

Polygalacturonases can be classified into two categories based on their mode of action: endopolygalacturonases (endoPGs) and exopolygalacturonases (exoPGs). EndoPGs (EC 3.2.1.15) act by the random cleavage of the homogalacturonan backbone of pectin internally, releasing oligogalacturonides (OGAs) as substrate [14,15,16]. ExoPGs (EC 3.2.1.67) act by the hydrolysis of the terminal residues of substrate polymers, releasing monomers [14,16,17]. Some PGs have also been shown to display both modes of action [16]. The role of endoPGs in plant pathogen interactions involves a complex cross-talk of signals and responses [13]. The oligogalacturonide (OGA) fragments released through the enzymatic activity of endoPGs can elicit a defense response by inducing the production of polygalacturonase-inhibiting proteins (PGIPs) in the plant, making the action of this enzyme a key step in the initial activation of plant defense mechanisms [18,19]. Furthermore, due to this interaction, endoPGs may also function as avirulence determinants [15,16,20] and will therefore serve as the focus of this study.

Many pathogen studies, including genome-wide studies, have been focused on in silico identification of effectors. However, in *Phytophthora* spp. only a few studies have identified PGs using bioinformatics tools [3,21]. A large family of PG genes has been isolated and characterized in *P. cinnamomi*, with more than 19 PG genes reported to be present in this pathogen [15]. Later, a review reported on the presence of 26 PG genes in *P. cinnamomi* using a bioinformatics approach [21]. Despite this knowledge, a comprehensive understanding and in-depth in silico identification and characterization of PGs in *P. cinnamomi* is lacking. Furthermore, limited studies are available on the functional characterization of PGs in *P. cinnamomi*. The lack of a reliable transformation system for *P. cinnamomi*, has posed a challenge for the functional characterization of genes in this pathogen. Therefore, elucidating the putative role of each gene encoding for PGs during plant-pathogen interactions through phylogenetic and expression analyses provides a framework for the selection of candidate virulence/pathogenicity genes in *P. cinnamomi* for future functional characterization.

The principles and techniques of phylogenetics for inferring relationships among genes, assessing homology and reconstructing evolutionary events provide a powerful tool to interpret sequence data [22]. The characterization and evolutionary analysis of PGs in *P. cinnamomi* was performed in 2002 by Götesson, A., Marshall, J.S., Jones, D.A. and Hardham, A.R. [15] before the release of the *P. cinnamomi* genome. Therefore, the divergence and evolution of these genes has not been investigated on a genome-wide scale. Furthermore, the functional role of *P. cinnamomi* PG genes based on inferred function of other *Phytophthora* spp. has not yet been investigated. The release and publication of several oomycete genomes has afforded an opportunity to investigate the evolutionary and phylogenetic relationships that exist between both *P. cinnamomi* PGs and those from other oomycete species [23,24,25,26,27,28].

The aim of this study was to identify endoPGs from the South African genome of *P. cinnamomi* involved in disease development in avocado. We identified and characterized endoPGs using in silico approaches from the genome sequence of a South African *P. cinnamomi* isolate [29]. Furthermore, we provide insight into the evolution of introns in *P. cinnamomi* endoPG genes and highlight putative PGs that may play an important role in infection. Future studies may be aimed at the functional characterization and targeted inhibition of these PG genes.

## 2. Materials and Methods

### 2.1. Identification of Full-Length P. cinnamomi PG Sequences

#### 2.1.1. Obtaining PG Sequences

PG sequences were obtained through a keyword search and the use of three different online database tools: National Center for Biotechnology Information (NCBI; https://www.ncbi.nlm.nih.gov (accessed on 11 July 2016)), the database for automated Carbohydrate-active enzyme Annotation (http://csbl.bmb.uga.edu/dbCAN/genome.php (accessed on 12 July 2016)) and the Joint Genome Institute (JGI) version (v) 1.0 (http://genome.jgi.doe.gov (accessed on 13 July 2016)) (Table S1).

#### 2.1.2. Identification of PG Sequences from the Genome of *P. cinnamomi*

To identify endoPGs from the South African *P. cinnamomi* genome, GKB4 accession number JAFJYM000000000 [29], two different approaches were used, namely hmmsearch and BLASTP analyses. PGs from the *Phytophthora* species (Table S1) were used to construct a multiple sequence alignment (MSA) using CLC Main Workbench v.8.0 (CLC bio, Cambridge, MA, USA). The MSA was used to generate a hmm profile, which was subsequently used to search for putative endoPGs from the *P. cinnamomi* genome. Additionally, a BLASTP analysis was performed against the generated protein database from the GKB4 genome using the PG protein sequences from other *Phytophthora* spp. (Table S1) as the query. Putative PGs from both analyses with a cut-off E-value of <1 × 10^−5^ were selected. Both analyses resulted in the same results, with differences in the E-value of the sequences. A BLASTP analysis against the non-redundant NCBI (NCBI-nr) database was performed to confirm endoPG sequences. EndoPG sequences were then renamed PcPG1 to PcPG37 from the results of the hmmsearch, based on the lowest to the highest E-values and deposited in the NCBI database with accessions: OL334941–OL334977.

#### 2.1.3. Identifying Full-Length and Partial PG Sequences

The full-length and partial endoPG sequences were grouped based on the presence or absence of the signature domains, respectively (Figure S1). The selection of full-length PGs was based on the length of the polypeptide chain, the presence of signature domains such as signal peptide, the GH 28 domain, catalytic and binding domains and the highly conserved cysteine rich N-terminal and C-terminal regions. The catalytic and binding domains and the cysteine residues were manually identified through a MSA using CLC Main Workbench v.8.1.2. The presence of the full N-terminal and C-terminal regions were assessed. The signal peptides were determined using SignalP v.4.1 [30] and the glycoside hydrolase (GH) 28 domain was identified using the hmmer online software, through the hmmscan tool (accessed on 12 June 2020; [31]).

### 2.2. Phylogenetic Tree Construction

Two separate phylogenetic trees were constructed. The first used a MSA consisting of identified full-length *P. cinnamomi* endoPG sequences from this study and full-length PG sequences from *Saprolegnia diclina*, and *Pythium vexans* as outgroups (Table S2). The second MSA contained full-length endoPG sequences from *P. cinnamomi*, other oomycete species (Table S3) and an outgroup species, *Fragilariopsis cylindrus* (Table S2). The MSAs were manually trimmed for phylogenetic tree construction and the best model that fits the data was selected using PartitionFinder 2 v.2.1.1 [32]. Phylogenetic trees were constructed using BEAST (Bayesian Evolutionary Analysis by Sampling Trees) v.2.4.8, Centre for Computational Evolution (Auckland, NZ) over a chain length of 50 million with sampling per 1000 states [33]. The parameters in BEAUti were set according to Drummond, A.J. and Bouckaert, R.R. [34] and used to generate the BEAST input file. The set parameters were as follows: WAG substitution model, gamma and invariant sites heterogeneity model, uncorrelated relaxed clock, speciation (Yule process tree prior), auto optimize operator and the rest of the parameters were set at default [34,35,36,37,38]. The BEAST output files from both trees were analyzed using Tracer v.1.6 (http://tree.bio.ed.ac.uk/software/tracer (accessed on 2 July 2020)). A 10% burn-in was removed from each tree using TreeAnnotator v.1.8.4, Drummond & Rambaut, (Auckland, NZ) and the annotated trees were visualized using Figtree v.1.4.3, Andrew Rambaut (Edinburgh, UK).

### 2.3. Identification of Intron/Exon Boundaries and Intron Loss/Gain Events

The intron/exon distribution patterns were predicted using Augustus (http://www.eumicrobedb.org/eumicrobedb/index.php (accessed on 10 July 2020)) and the selected *P. cinnamomi* species model. cDNA and genomic clones of each endoPG gene containing introns were isolated and sequenced. The genomic and coding sequences were aligned using CLC Main Workbench to confirm the intron/exon boundaries (Figures S2 and S3). Coding sequences were obtained through a PCR amplification of cDNA prepared previously, as a template. Primer pairs were designed for each individual endoPG predicted to contain introns, to amplify the entire coding sequence (Table 1). The PCR fragments were purified using the Zymoclean Gel DNA Recovery Kit (Zymo Research, Orange, CA, USA) according to the manufacturer’s instructions and subsequently cloned into a pMiniT™ 2.0 *Escherichia coli* plasmid cloning vector (NEB™ PCR Cloning Kit; Competent cells: *E. coli* DH5α, Vector: pMiniT™ 2.0; Biolabs, Cambridge, MA, USA). This was followed by colony PCR using vector specific primers and sequencing of the PCR products. The sequences obtained from sequencing using the forward and reverse primers were assembled into a contig and analyzed as described above using CLC Main Workbench.

To analyze the evolution of introns from each endoPG gene, the gene structure predicted (Table S4) was aligned to the corresponding endoPG protein sequence on the phylogenetic tree. Intron loss-and-gain events were determined based on the divergence of the genes according to the phylogenetic tree.

### 2.4. Inoculation of Avocado Rootstocks and Harvesting of Infected Root Samples

RNA-seq data from an experiment performed previously (unpublished data) were used for expression analysis in this study. *P. cinnamomi* isolate GKB4 was used to infect a susceptible avocado rootstock, R0.12. Briefly, zoospores were produced according to the modified protocol of Chen, D.-W. and Zentmyer, G. [39] and inoculation was carried out immediately after the zoospore collection to ensure motility of the zoospores. Plants were inoculated by soaking the roots in a zoospore suspension of 1.4 × 10^5^ zoospores/mL for 2 h. Root material was harvested from the infected R0.12 plantlets at 12 h post inoculation (hpi), 24 hpi and 120 hpi, snap frozen in liquid nitrogen and stored at −80 °C until use. Three biological replicates were collected per timepoint and each biological replicate contained three plants. In a separate experiment, a culture of *P. cinnamomi* GKB4 was grown on V8 media (200 mL clarified V8 juice, 2 g CaCO_3_, 15 g agar and distilled water to a volume of 1 L) and incubated for two weeks to serve as a control.

### 2.5. RNA Extraction, Sequencing and Differential Gene Expression Analysis of P. cinnamomi PG Genes during Avocado Infection

Total RNA was extracted from finely-ground infected avocado root tissue and *P. cinnamomi* mycelia, using the modified cetyl-trimethyl-ammonium-bromide (CTAB) lithium chloride protocol [40]. RNA samples were treated with RNase-free DNase 1 (Fermentas, Life Sciences, Hanover, NH, USA) and purified using the Qiagen RNeasy clean-up kit (Qiagen, Valencia, CA, USA). RNA concentration and integrity was determined using a NanoDrop 2000 Spectrophotometer (Thermo Scientific, Wilmington, DE, USA) and 2% non-denaturing TAE agarose gels. RNA quality was assessed using the Agilent 2100 Bioanalyzer (Agilent Technologies, Santa Clara, CA, USA) and sent to Novogene (Novogene Corporation Inc., Cambridge, UK) for sequencing. Sequencing was performed for RNA samples extracted from the diseased susceptible avocado rootstock at 12 hpi, 24 hpi and 120 hpi and mycelia grown in culture. Three biological replicates of each sample were sequenced using the Illumina Hiseq PE150 sequencing platform. Illumina paired-end reads were trimmed using Trimmomatic v.0.39, Bolger AM, Lohse M, Usadel M, AM Muhlenberg [41]. Quality control analysis of the raw reads was conducted using FASTQC and summarized using MultiQC v.1.9, Ewels P, (Stockholm, Swedan) [42]. RNA-seq reads were aligned to the genome of *P. cinnamomi* using HISAT v.2.0.6, Kim D, (TX, USA) ([43] and reads that mapped uniquely to the genome were selected for further analyses. Splice junctions with a high confidence level were determined using Portcullis v1.2.0, Earlham institute, (Norwich, UK) [44]. The trimmed reads were aligned again using HISAT2, informed by the high-confidence splice junctions obtained from Portcullis. Raw read counts at a gene level were obtained from reads aligning to exons using HTSeq v.0.13.5, Putri G, Anders S, Zanini F, (Kensington, Australia) [45]. Raw read counts were normalized and analyzed using DESeq2 v.2.0.1, Love MI, Anders S, Huber W, (Heidelberg, Germany) [46].

## 3. Results

### 3.1. Genome-Wide Identification of P. cinnamomi endoPGs

A total of 37 putative *P. cinnamomi* PG (PcPG) sequences (PcPG1 to PcPG37) were identified (Table 2) and confirmed as endoPGs based on a BLASTP analysis against the non-redundant NCBI-nr database (Table S5).

### 3.2. Full-Length Protein and Signature Domain Analysis

Of the 37 endoPGs from *P. cinnamomi*, 19 full-length and 18 partial sequences were identified. The full-length sequences contained polypeptide chains ranging from 328 to 524 amino acids (aa), and signal peptides ranging from 19–23 aa. The sequences also contained the glycoside hydrolase 28 (GH28) domain (Table S6) and four highly conserved catalytic (S/GHG) and binding (NTD and RIK) domains associated with PGs. Furthermore, the sequences contained the invariantly conserved Y residue and the highly conserved cysteine rich regions (Figure S4).

Based on the endoPG sequence of PcPG6, the binding domains were in positions N^177^ T^178^ D^179^ and R^258^ I^259^ K^260^ and the catalytic domains were in positions Q^199^ D^200^ D^201^ and G^221^ H^222^ G^223^. The invariantly conserved residue Y^299^ was observed in all full-length PG sequences. The disulphide bridges in the N-terminal and C-terminal regions were found in positions C^33^, C^48^ and C^202^, C^218^, respectively. An additional disulphide bridge was observed between C^353^ and C^362^, which was also found in PcPG7, PcPG8, PcPG9, PcPG11, PcPG12, PcPG13, PcPG14, PcPG16, PcPG17, PcPG19, PcPG22 and PcPG25 (Figure S4). Furthermore, PcPG23 lacked a short region in the C-terminal end, rendering the protein sequence incomplete. The 18 partial endoPG sequences identified in this study were considered as pseudogenes.

### 3.3. Evolution of PGs in P. cinnamomi

**Figure 1:**
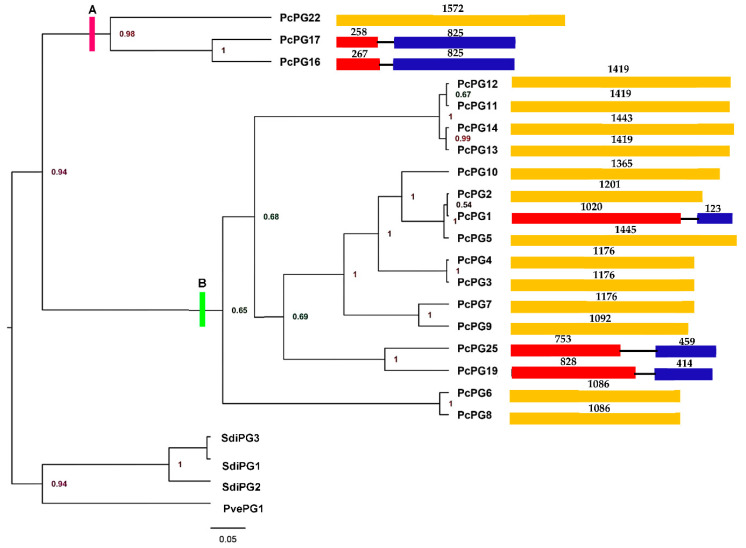
Phylogenetic tree and gene structure analysis of 19 full-length PGs from *Phytophthora cinnamomi* (Pc) with PGs from *Saprolegnia diclina* (Sdi) and *Pythium vexans* (Pv) species as outgroups. The tree was constructed using a Bayesian inference method implemented in BEAUti over a chain length of 50 million with sampling per 1000 states. The numbers in the nodes represent the posterior probabilities. Only posterior probability values > 0.9 were considered. PcPGs grouped into two distinct clades, Clade A (purple line partition) and Clade B (green line partition). In the gene structures, exons are represented by rectangles and introns by black lines. The numbers above the gene structures indicate the size of the exon regions in bp.

Endopolygalacturonases from *P. cinnamomi* grouped into two distinct clades, here labelled Clades A and B (Figure 1), with a posterior probability score of 0.94. Most PG genes from *P. cinnamomi* were intronless (*pcpg2* to *pcpg14*, and *pcpg22*). Additionally, five PcPG genes (*pcpg1*, *pcpg16*, *pcpg17*, *pcpg19* and *pcpg25*) were interrupted by an intron. The introns of genes in Clade A, and in *pcpg19* and *pcpg25* in Clade B, were unique but located in the same position (Figure 1). Furthermore, the intron in *pcpg1* (Clade B) was in a different position and unique.

### 3.4. Phylogenetic Relationship between endoPGs from P. cinnamomi and Other Oomycetes

The PGs from *P. cinnamomi* were scattered in the phylogenetic tree and clustered into different clades. A close relationship was observed for PcPG17 to the uncharacterized PG2 from *Phytophthora sojae*. Some PGs from *P. cinnamomi* were also shown to be very closely related to each other, such as PcPG7 and PcPG9, PcPG1 and PcPG2, PcPG11 and PcPG12 and PcPG13 and PcPG14. The distribution of PcPGs in different groups in the phylogenetic tree constructed assisted in inferring putative function to PcPGs, based on sequence similarity. PcPG19 and PcPG25 clustered with *P. parasitica* PG10 (PpPG10) with a similarity of 68.77 and 59.63%, respectively. These two PGs also formed a cluster with the characterized PG1 from *P. infestans* (PiPG1) and PpPG1 with similarity scores of 70.53 and 68.77% for PcPG19 and 68.90 and 55.37% for PcPG25, respectively. PcPG3, PcPG4, PcPG7 and PcPG9 formed a cluster with PpPG2 from *P. parasitica* with similarity scores of 84.14, 84.14, 79.23 and 74.41%, respectively.

**Figure 2:**
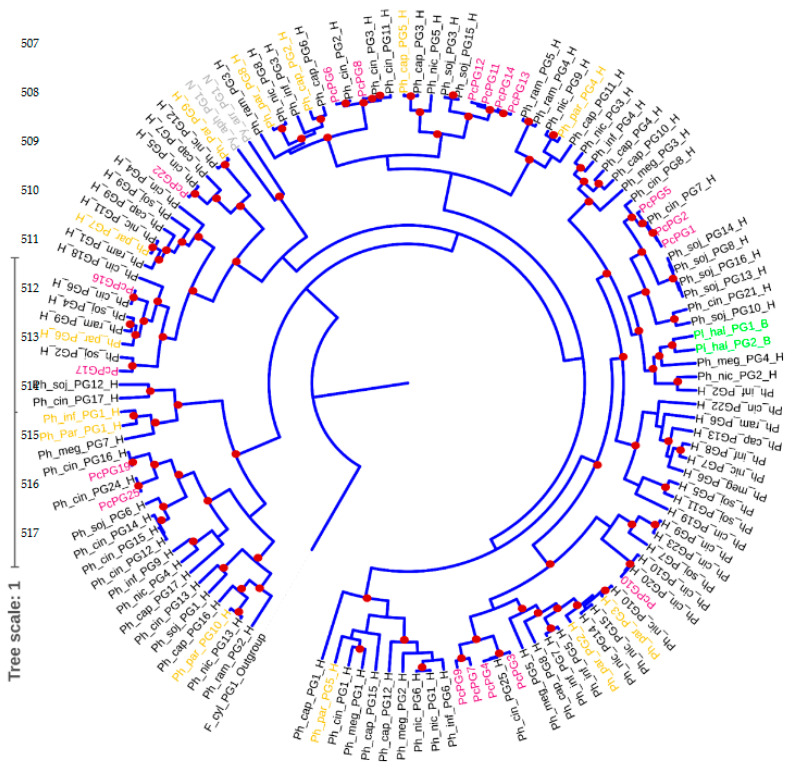
Phylogenetic analysis of PGs from oomycete species. The phylogenetic tree was constructed using BEAST over a chain length of 50 million and sampling per 1000 states. Significant posterior probabilities (>0.95) are indicated by red dots on the nodes. Oomycetes PGs with different trophic lifestyles, B—biotrophic, H—hemibiotrophic, N—necrotrophic, were used for the phylogenetic tree construction, with *Fragilariopsis cylindrus* as an outgroup. PG sequences from *Phytophthora cinnamomi* identified in this study are highlighted in purple. Characterized PG sequences from *Phytophthora* spp. are highlighted in orange. The PGs from biotrophic and necrotrophic species are highlighted in green and grey, respectively.

PcPG6 and PcPG8 clustered with PpPG8 from *P. parasitica* with similarity scores of 92.24 and 91.97%, respectively. These two proteins also clustered with the characterized protein PciPG2 from *Phytophthora capsica* with similarity scores of 81.98% for PcPG6 and 80.11% for PcPG8. PcPG16 and PcPG17 clustered with PpPG6 from *P. parasitica* with similarity scores of 93.39 and 83.75%, respectively. PcPG22 showed a close relation to PpPG9 from *P. parasitica* with a similarity score of 79.75% and PcPG11, PcPG12, PcPG13 and PcPG14 clustered with PpPG5 from *P. parasitica* with a similarity score of 69.03, 69.03, 68.84 and 68.73%, respectively. Lastly, PcPG1, PcPG2 and PcPG5 formed a cluster with PGs from the biotrophic pathogen, *Plasmopara halstedii* (Figure 2).

### 3.5. Time-Course Gene Expression of PG Genes from P. cinnamomi during Colonization of a Susceptible Avocado Rootstock

**Figure 3:**
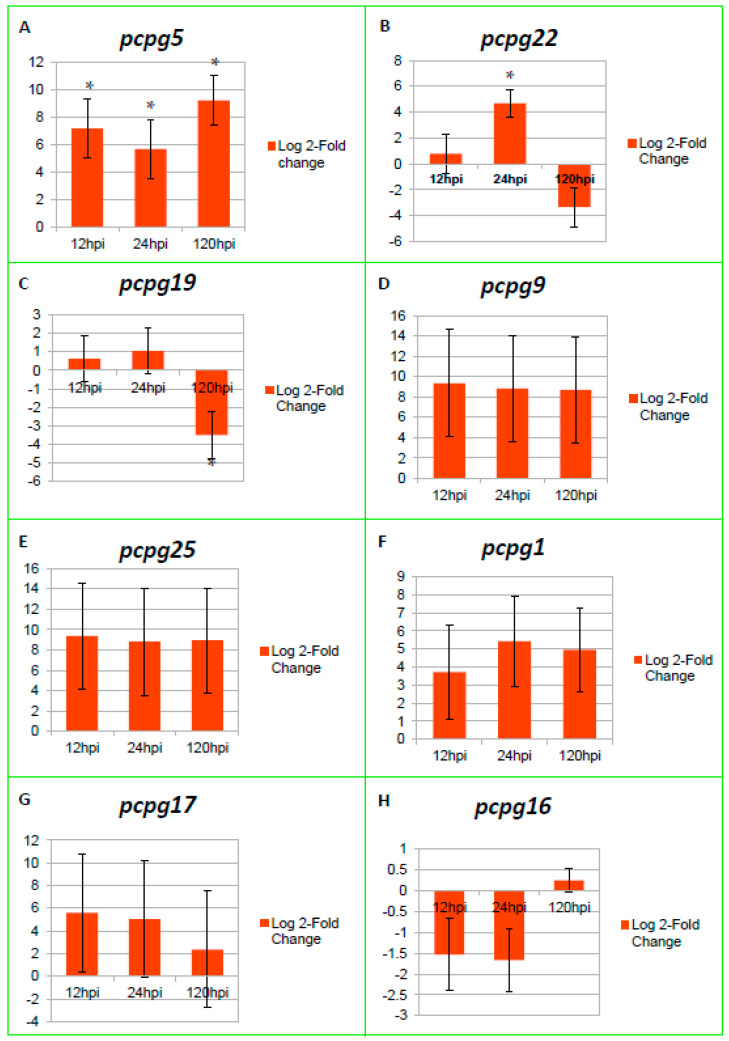
Time-course gene expression of eight PcPG genes expressed during colonization of a susceptible R0.12 avocado rootstock by *Phytophthora cinnamomi*. The Y-axis represents the log 2-Fold Change expression of (**A**) *pcpg5*, (**B**) *pcpg22*, (**C**) *pcpg19*, (**D**) *pcpg9*, (**E**) *pcpg25,* (**F**) *pcpg1,* (**G**) *pcpg17,* (**H**) *pcpg16*, and the X-axis represents the different timepoints at which each gene is expressed during colonization. Expression profiles were generated from RNA-seq data obtained from infected susceptible R0.12 avocado roots harvested at 12 hpi, 24 hpi and 120 hpi. PG gene expression is indicated by a vertical bar, with error bars indicating standard error (SE). Statistical significance of PG expression was calculated using DESeq2, based on the read counts from the RNA seq data. Significant expression relative to mycelia at a p-adj-value of <0.05 is indicated by a single asterisk above/below each error bar. Log 2-Fold Change >1 represents up-regulated genes and log 2-Fold Change > −1 represents down-regulated genes.

A total of eight PcPG genes (*pcpg1*, *pcpg5*, *pcpg9*, *pcpg16*, *pcpg17*, *pcpg19*, *pcpg22* and *pcpg25*) were expressed by *P. cinnamomi* with only seven genes showing induction in planta (p-adj ≤ 0.5 and log 2-Fold Change (FC) >1 (up-regulated) and log 2-FC > −1 down-regulated). However, only three genes (*pcpg5*, *pcpg22* and *pcpg19*) were significantly up- or down-regulated when compared to the mycelial control during the early and late stage of infection, respectively (Figure 3A–C; Table S7). In this study, 12 hpi and 24 hpi represent the biotrophic phase of infection when the roots are still healthy, whereas, the necrotrophic phase is represented by 120 hpi when the roots are dead. *Pcpg5* was significantly up-regulated at all three timepoints, with peak expression observed at 120 hpi (9.25-log 2-FC; Figure 3A). Similarly, *pcpg1*, *pcpg9* and *pcpg25* were induced at very high levels during the time course of infection, with peak expression observed at 12 hpi (9.36-log 2-FC; 9.36-log 2-FC) for *pcpg9* and *pcpg25*, respectively, and at 24 hpi (5.43-log 2-FC) for *pcpg1*. *Pcpg22* expression was similar to that of mycelia at 12 hpi (0.79-log 2-FC), followed by a significant increase at 24 hpi (4.68-log 2-FC), which sharply declined by 120 hpi (−3.34-log 2-FC) (Figure 3B). Additionally, expression of *pcpg19* remained similar to that of mycelia at 12 hpi (0.62-log 2-FC) and 24 hpi (1.05-log 2-FC), followed by significant down-regulation at 120 hpi (−3.50-log 2-FC; Figure 3C). A progressive decline in gene expression was observed for *pcpg17* at all timepoints, with peak expression, although not significant, at 12 hpi (5.58-log 2-FC). *Pcpg16* was down-regulated at 12 hpi (−1.53-log 2-FC) and 24 hpi (−1.67-log 2-FC) followed by a very low increase in gene expression similar to that of mycelia, at 120 hpi (0.24-log 2-FC). 

## 4. Discussion

In this study we identified and characterized a repertoire of PGs from the genome of a South African isolate of *P. cinnamomi*. Using BLASTP and hmmsearch analyses, we identified a large family of 37 endoPGs. The utilization of the high-quality genome sequence of *P. cinnamomi* resulted in the identification of more PGs than previously reported for the pathogen [3,15]. Large PG families have also been found in other *Phytophthora* spp. [3,16,47]. Based on deposited sequence data from online databases, a total of 21 PGs were found in *P. capsici*, 19 in *P. infestans*, 16 in *Phytophthora ramorum* and 25 in *P. sojae* (Table S1).

The biological significance of having numerous PGs present is unclear, but several explanations have been proposed [15]. The initial proposal suggested that the number of PG genes present in a pathogen is correlated to the pathogen’s host range. Pathogens with a wide host range have a large PG gene family and those with a narrow host range have a small PG gene family [19]. This may very well be the case as both *P. cinnamomi* and *P. parasitica* have a wide host range and a large PG gene family. However, species such as *P. sojae* and *P. infestans* have a large gene family and narrow host range. Therefore, factors other than the host range, such as plant cell wall complexity, composition and structure [48], could provide selective pressures that lead to the evolution of larger PG gene families [15]. Furthermore, in order to degrade the complex structure of the plant cell wall and in turn gain nutrients from the degradation products, the large PG gene family will allow the pathogen to employ different PGs with different specificities and affinity for the substrate for successful penetration and colonization of the host plant. Therefore, the continuous evolutionary arms race between a pathogen and its host plant involving virulence factors from the pathogen and their targets in the host may also contribute to the large number of PG families observed in *P. cinnamomi*. The pathogen produces these enzymes in response to the need to circumvent inhibition by polygalacturonase-inhibiting proteins secreted by the host and other defense responses the pathogen may encounter in different hosts, in order to establish successful colonization of the host [15,49].

Endopolygalacturonase genes in *Phytophthora* spp. diverged prior to speciation and can thus still undergo duplication after this speciation event, leading to large endoPG gene families being formed in these pathogens [47]. Therefore, we propose that the large PG gene family identified in the South African genome of *P. cinnamomi* may also be as a result of gene duplication events occurring after its speciation. Furthermore, the evolution of the *P. cinnamomi* PG multigene family has previously been reported by Götesson, A., Marshall, J.S., Jones, D.A. and Hardham, A.R. [15] as a result of birth-and-death evolution, reticulate evolution and adaptive evolution.

As expected, all full-length PcPGs in this study contained the highly conserved S/GHG domain [13]. The H residue in this domain is responsible for maintaining ionization, as it serves as a proton donor in the catalytic site of PGs, making this residue important for enzyme catalysis [50,51]. Three strictly conserved D residues from the NTD and QDD domains play a key role in catalysis, serving as the primary active site component in PGs from several *Phytophthora* spp. [14,15]. The highly conserved NTD and RIK domains present in all full-length PcPGs are important for binding to the substrate [50,52]. All full-length PcPGs contained two to three disulphide bridges, which is expected as based on the crystal structure of PGs; these enzymes usually contain two, sometimes three, disulphide bridges that stabilize the molecules [53]. The significance of the additional disulphide bridge present in some PcPGs is unclear. However, we propose that it may contribute to increased stability of the protein or the architecture of the protein may require this disulphide bridge for functionality. This provides strong evidence that the full-length PcPGs identified in this study have the potential to be fully functional when in contact with the host plant. Although we report on 18 non-functional PGs that are considered pseudogenes in this study, there is a possibility that some genes may be functional and may have been missed in this study due to possible errors in the annotation of the *P. cinnamomi* genome used in this study. Therefore, further experimental validation of these genes should be performed.

Only three PG genes out of the eight expressed PG genes, were significantly expressed during the colonization of the susceptible avocado rootstock. One contributing factor to the observed results is due to the low read counts of the pathogen observed in the RNA-seq data. The observed expression pattern of PcPG genes in planta enables us to make predictions about the possible contributions of individual genes to virulence of *P. cinnamomi*. Significantly higher expression of *pcpg5* when compared with the mycelial control across all timepoints suggests that PcPG5 may lead to the release of pectin degradation substrates that subsequently induce the expression of other genes encoding PGs in *P. cinnamomi* [54]. Therefore, this gene may be important during the early stages of infection (biotrophy) to release polymers such as galacturonic acid, triggering the synthesis of additional PGs such as *pcpg22*, *pcpg9*, *pcpg25*, *pcpg1* and *pcpg17* that are inducible in the *P. cinnamomi*-avocado interaction [55]. Similar to the gene expression of *pcpg5*, *pg1* from *Fusarium oxysporum* was expressed during the entire disease cycle, with optimal expression observed during the final disease stages [56]. Additionally, *Bcg1* from *Botrytis cinerea* was constitutively expressed during infection of tomato leaves [57]. The protein encoding for this gene is required for full virulence on tomato, apple and broad bean [8,57].

PG genes from *P. cinnamomi* were induced or repressed during the different stages of the pathogen’s lifecycle. Similarly, in *B. cinerea*, differential gene expression of endoPG genes was observed in various stages of infection [57]. Furthermore, a sequential transcription of genes was observed in PG genes from *Sclerotinia sclerotiorum* [58]. Significantly higher and lower expression was observed for *pcpg22* at 24 hpi and *pcpg19* at 120 hpi when compared with the mycelium in vitro expression, respectively. The significant expression of *pcpg22* during the early phase of infection was similar to the expression profile of *pg5* from *F. oxysporum,* which was expressed during the initial stages of infection of tomato [59]. However, this gene was not required for pathogenicity of *F. oxysporum* on tomato plants or for invasive growth on living plant tissue. Additionally, *Bcpg2* from *B. cinerea* and *sspg2* from *S. sclerotiorum* were highly expressed during the early hours of infection of tomato and sunflower cotyledons, respectively, with decreased expression at the late infection stage [57,60]. Furthermore, *pcpg9*, *pcpg25*, *pcpg1* and *pcpg17* were highly induced during the biotrophic phase of infection. This suggests that the pathogen employs *pcpg1*, *pcpg5, pcpg9, pcpg19, pcpg17, pcpg22* and *pcpg25* during the early stage of infection, then down-regulates *pcpg22* and *pcpg19*, allowing the pathogen to only utilize *pcpg5*, *pcpg1*, *pcpg9*, *pcpg17* and *pcpg25* at 120 hpi during necrotrophy. Therefore, these observed expression patterns suggest the involvement of *pcpg5* and *pcpg22* in penetration during the biotrophic phase of infection and *pcpg5* in tissue maceration during the necrotrophic phase of infection. The significant down-regulation of *pcpg19* at 120 hpi suggests that this gene does not play a critical role in infection during the necrotrophic phase and may be involved in symptom development during the biotrophic phase of infection. The induction of *pcpg16* in planta was very low, suggesting that this gene does not play a role in pathogenesis during the *P. cinnamomi*-avocado interaction. Although *pcpg1*, *pcpg9*, *pcpg17* and *pcpg25* were not significantly expressed, we suspect that these genes may be playing a critical role in the biotrophic phase of infection, due to their induced expression at very high levels in planta (Figure 3), during the early stages of infection. Furthermore, these PGs may also play a crucial role in the induction of disease symptoms on infected susceptible avocado plants. The potential role of these genes in penetration and early colonization warrants further investigation.

The products released at each stage during the degradation of pectin, starting with the release of oligomers from the initial degradation by endoPGs secreted early in infection, affect the successive induction and repression of PG genes and encode for PGs with different biochemical properties until complete degradation of pectin has been reached [15,61]. The proteins derived from these PcPG genes may have different substrate specificity, pH optimum and enzyme activity, providing an advantage to the pathogen as explained in Götesson, A., Marshall, J.S., Jones, D.A. and Hardham, A.R. [15]. Therefore, the differential up- and down-regulation of PcPG genes may increase the virulence of *P. cinnamomi* through the efficient degradation of the plant cell wall and subsequent nutrient acquisition.

The majority of the PG genes (six) were up-regulated during the early stages of plant infection, whereas only one PG (*pcpg5*) was induced during the necrotrophic phase of infection. *P. cinnamomi* follows a hemibiotrophic lifestyle, whereby the pathogen initially infects the susceptible avocado roots as a biotroph, then switches to a necrotrophic infection strategy. Biotrophs evade or suppress host defense to obtain nutrients from living plant tissue [62,63]. Necrotrophic pathogens acquire carbon and energy through the extensive degradation of plant tissue, leading to the eventual death of the host plant [64]. Based on the observed results from this study, we postulate that *P. cinnamomi* may be secreting a limited number of PGs (six) during the biotrophic phase (12 hpi and 24 hpi) of infection to evade host defense responses. Interestingly, only one PG gene was significantly up-regulated during the necrotrophic phase (120 hpi) of infection. Considering the infection strategy of the necrotrophic pathogens, we expected to observe a large number of PG genes expressed at the later timepoints [65]. The observed phenomena may suggest that in the *P. cinnamomi*-avocado interaction, PGs play a significant role in the early stage of pathogen penetration and colonization; meanwhile, only PcPG5 may be required by the pathogen during the late infection stage when colonization has been established. However, it is worth noting that *pcpg1*, *pcpg9*, *pcpg17* and *pcpg25*, although not significantly expressed, may also be involved in tissue maceration and cause observable symptoms in the susceptible avocado rootstock during the necrotrophic phase. Alternatively, the pathogen may employ other cell wall degrading enzymes in addition to PcPG5 during the late infection stage.

The distribution of PGs from *P. cinnamomi* showed that only PcPG17 was closely related to PG2 from *P. sojae*, suggesting that the genes encoding these proteins are orthologs and may have evolved prior to speciation [16]. Similarly, most of the PGs from *Aspergillus* were closely related to PGs from other species [66], supporting the suggestion that PGs diversified prior to species divergence. Contrary to this observation, a close relation was observed for PcPG7 and PcPG9, PcPG1 and PcPG2, PcPG11 and PcPG12, and PcPG13 and PcPG14, suggesting that the genes encoding these proteins may have undergone recent gene duplication events. Similar results have been observed for *pppg2* and *pppg3* genes from *P. parasitica* [47]. This phenomenon has also been observed in other secretory proteins of *Phytophthora* spp. [47,67]. Therefore, the current study supports the previous reports suggesting that the endoPG genes diverged prior to speciation within the genus of *Phytophthora* and that certain endoPG genes can still undergo duplication after this speciation event, resulting in the formation of large endoPG gene families [47].

To study the functional characteristics of PG proteins from *P. cinnamomi*, we inferred putative function based on the relatedness of PcPGs to PGs from other *Phytophthora* spp. that have been assigned function. This clustering highlighted the possibility that PGs may play an important role in infection and may affect plant physiology and growth. PcPG19 and PcPG25 clustered with *P. parasitica* PG10 (PpPG10), which causes leaf yellowing and curling as well as dwarfism of *Nicotiana benthamiana* [47]. These two PGs also formed a cluster with PG1 from *P. infestans* (PiPG1) and PpPG1. Notably, *pppg1* was up-regulated in tomato leaves during the early phase of infection [16].

Similarly, *pipg1* was expressed in pre-infection and early infection stages suggesting a role in penetration and invasion of plant tissue [13]. Therefore, the clustering of PcPG19 suggests a possible role of this gene in the early biotrophic phase of infection in cell wall penetration of the avocado plant and in virulence of the pathogen. PcPG25 may be responsible for causing yellowing, leaf curling and dwarfism of avocado plants. PcPG3, PcPG4, PcPG7 and PcPG9 clustered with PpPG2, which is associated with yellowing and dwarfism in tobacco plants [47]. The clustering of PcPG9 with PpPG2 from *P. parasitica*, suggests the possible role of PcPG9 in yellowing and dwarfism of susceptible avocado plants. Similar symptoms were observed for tobacco plants expressing *pppg8*, whose protein clustered with PcPG6 and PcPG8 [47]. These two proteins also clustered with *Phytophthora capsici* PG2 (PciPG2). *Pcipg2* was strongly up-regulated in pepper leaves and caused necrotic spots [14]. PcPG16 and PcPg17 clustered with PpPG6; *pppg6* is associated with dwarfism, curling and stunted growth of new leaves and wrinkling with necrotic spots on expanded leaves in tobacco plants expressing this gene [47]. These symptoms may be caused by PcPG17 during all three timepoints of infection in the susceptible avocado plant. PcPG22 was closely related to PpPG9 while PcPG11, PcPG12, PcPG13 and PcPG14 clustered with PpPG5. *Pppg5* expression in transgenic tobacco caused silvery leaves and deformed mesophyll cells when infected with *P. parasitica* [47]. PcPG1, PcPG2 and PcPG5 formed a cluster with PGs from the biotroph *Plasmopara halstedii* (Figure 2). The clustering of PcPG5 with PGs from the biotrophic species *P. halstedii* supports a possible role in the early biotrophic stage of infection as indicated by the expression data.

The PcPGs that were not expressed during colonization of a susceptible avocado rootstock at the timepoints selected in this study, but formed clusters with characterized PGs from other oomycete species, may function in this pathosystem. However, the conditions and time of harvesting in our experimental design may not be ideal for capturing the expression of these PG genes. Alternatively, the proteins derived from these genes may not be functional at all in establishing infection in avocado. Furthermore, the full-length PG genes not expressed in this study may play a critical role in the penetration and colonization of other host plants of *P. cinnamomi*, considering that the pathogen has a wide host range of more than 5000 plant species [21]. A study conducted by ten Have, A., Breuil, WO, Wubben, J.P., Visser, J. and van Kan, J.A. [57] showed that the expression of *Bcpg* gene family in planta is differential and dependent on the host tissue, the stage of infection and temperature.

The majority of PcPGs that clustered into Clade A had an intron in their nuclear genes, while most genes representative of Clade B were intronless. The intron sequences were present at varying positions in some genes of both Clade A and Clade B (Figure 1). This suggests two possibilities, namely (i) that the ancestral *P. cinnamomi* PG gene was intronless and that introns were gained separately for both Clade A and B or (ii) that the ancestral *P. cinnamomi* PG gene had one or more introns that were differentially lost. 

The evolution of introns is a dynamic process in eukaryotes, and introns can be acquired by or eliminated from a gene during evolution [68,69]. The ancestral state of PcPG genes is unknown. However, due to the presence and absence of introns, their uniqueness and location in different positions, we suggest that over evolutionary time intron gain and loss events occurred. Similarly, J-S Hong, K-H Ryu, S-J Kwon, J-W Kim, K-S Kim and K-C Park [66] proposed that intron gain and loss events might have occurred, which has led to the PGs present in *Aspergillus* and *Neurospora crassa*. Furthermore, the loss and gain of introns in different genomes are as a result of strong selective pressures contributing to genome adaptation [69]. Therefore, the gain and loss of introns in PcPG genes may be important to confer fitness to the pathogen and corresponds to the later synthetic theory of intron evolution which combines both “intron late” and “intron early” theories [70]. 

## 5. Conclusions

Previous studies have identified PGs in *P. cinnamomi*, however, genome-wide characterization of these genes has not been performed. Therefore, this study provides the first report on the in silico identification and characterization of PG sequences from the genome of a South African *P. cinnamomi* isolate, GKB4. The study shows that *P. cinnamomi* harbors more PG genes in its genome than previously reported. Furthermore, this is the first study that has identified candidate PGs from *P. cinnamomi* and showed that many are induced during the early infection stage of avocado. We identified 37 PG sequences, of which 19 were full-length proteins. Inferred putative function was assigned to eight PcPGs based on relations to characterized PGs from other *Phytophthora* spp. and the gene expression profile during colonization of the susceptible avocado rootstock in this study, informed by the literature. Nine PcPGs clustered with PGs from other oomycete species that have been characterized, indicating a possible function of these enzymes. However, based on the fact that the genes encoding these enzymes were not expressed in our pathosystem, this suggests a possible role of the PGs in infection or symptom development in other host plants of *P. cinnamomi*, or they may be functional in other timepoints not investigated in this study.

The functional characterization of PG genes in *P. cinnamomi* is lacking, due to the need of a reliable transformation system. Although this is the case, this study highlighted candidate pathogenicity/virulence PGs from *P. cinnamomi* that may play a critical role during the infection of avocado. Therefore, in this study, we have provided a framework for future functional characterization of these genes in order to investigate the exact role these enzymes play in avocado cell wall degradation and their interaction with PG inhibitor proteins from avocado once a reliable transformation system is established. We also showed that over evolutionary time, there have been both intron gains and losses in PGs from *P. cinnamomi* contributing to the pathogen’s fitness. Moreover, due to the synteny that exists between *Phytophthora* spp., the results from this study can be applied to studies of other *Phytophthora* spp. as a result of the potential conservation of pathogenicity features between *Phytophthora* species. Overall, this study provides knowledge on the mechanisms employed by the pathogen for infection and disease development which will aid in the better management of the disease.

## Figures and Tables

**Table 1:** Primer pairs designed for the coding sequences used for amplification of each endoPG from *P. cinnamomi*.

Gene Coding Sequence	Forward Primer (5′-3′)	Reverse Primer (5′-3′)
*pcpg1*	ATGAAGTTCTTCACCACTGCG	CTACACGCTCATGCTGTTGG
*pcpg16*	ATGAAGTTCTTCGCCCCCGTCC	TTAGCAGTCCACGCTGCTGGG
*pcpg17*	ATGAAGTTCTTCGCCCCCGTCC	TTAGCAAGACACTCCGCTCGGTTC
*pcpg19*	ATGAAGGCTTTCTCCGCTCTC	CTTAGCACGGGACATTGGAC
*pcpg25*	ATGAAGTTTTTCTCCGCCTTATTCAC	TTAGCACGGGACATTGTACGG

**Table 2:** Results of the endopolygalacturonase proteins from the hmmsearch and BLASTP analyses.

Sequence NCBI ID	Renamed Sequence	Hmmsearch E-Value ^a^	Hmmscan Score ^b^	BLASTP E-Value ^a^	BLASTP Score ^b^
OL334941	PcPG1	2.4 × 10^−196^	652.2	1 × 10^−159^	450
OL334942	PcPG2	3.8 × 10^−196^	651.5	1 × 10^−155^	441
OL334943	PcPG3	5.1 × 10^−196^	651.1	1 × 10^−157^	446
OL334944	PcPG4	5.1 × 10^−196^	651.1	1 × 10^−157^	446
OL334945	PcPG5	1.1 × 10^−195^	650.0	1 × 10^−153^	436
OL334946	PcPG6	3.5 × 10^−195^	648.4	1 × 10^−154^	436
OL334947	PcPG7	4.2 × 10^−195^	648.1	1 × 10^−156^	444
OL334948	PcPG8	8.5 × 10^−195^	647.1	1 × 10^−158^	447
OL334949	PcPG9	1.1 × 10^−194^	646.7	1 × 10^−156^	442
OL334950	PcPG10	3.7 × 10^−192^	638.4	1 × 10^−155^	444
OL334951	PcPG11	2.5 × 10^−190^	632.4	1 × 10^−159^	453
OL334952	PcPG12	2.5 × 10^−190^	632.4	1 × 10^−159^	453
OL334953	PcPG13	1.6 × 10^−189^	629.7	1 × 10^−157^	450
OL334954	PcPG14	2 × 10^−189^	629.4	1 × 10^−158^	451
OL334955	PcPG15	1.3 × 10^−187^	623.5	1 × 10^−163^	460
OL334956	PcPG16	2.4 × 10^−185^	616.0	1 × 10^−150^	427
OL334957	PcPG17	2.1 × 10^−183^	609.6	1 × 10^−139^	398
OL334958	PcPG18	1.9 × 10^−177^	590.0	0.0	537
OL334959	PcPG19	5.8 × 10^−176^	585.1	1 × 10^−120^	352
OL334960	PcPG20	1.4 × 10^−175^	583.8	1 × 10^−130^	376
OL334961	PcPG21	1 × 10^−169^	564.6	1 × 10^−136^	390
OL334962	PcPG22	4.7 × 10^−168^	559.1	1 × 10^−120^	367
OL334963	PcPG23	2.8 × 10^−164^	546.7	1 × 10^−136^	392
OL334964	PcPG24	1.3 × 10^−161^	537.9	1 × 10^−130^	369
OL334965	PcPG25	7.2 × 10^−155^	515.7	1 × 10^−107^	319
OL334966	PcPG26	1.5 × 10^−98^	330.4	1 × 10^−71^	221
OL334967	PcPG27	3.2 × 10^−91^	306.4	5 × 10^−79^	242
OL334968	PcPG28	7 × 10^−86^	288.8	7 × 10^−66^	206
OL334969	PcPG29	5.1 × 10^−82^	276.1	1 × 10^−57^	184
OL334970	PcPG30	1.9 × 10^−81^	274.2	8 × 10^−55^	176
OL334971	PcPG31	6.4 × 10^−78^	262.6	6 × 10^−63^	198
OL334972	PcPG32	3.3 × 10^−71^	240.5	7 × 10^−63^	197
OL334973	PcPG33	5.7 × 10^−62^	210.1	1 × 10^−52^	171
OL334974	PcPG34	2.8 × 10^−53^	181.5	2 × 10^−51^	167
OL334975	PcPG35	2 × 10^−31^	109.7	5 × 10^−23^	94
OL334976	PcPG36	5.1 × 10^−24^	85.3	7 × 10^−17^	74
OL334977	PcPG37	6.6 × 10^−14^	52.0	1 × 10^−7^	49

^a^ Statistical significance × 10 of the match to the database sequence. A lower E-value represents higher significance. ^b^ Bit score, which is the log-odds score for the complete sequence. Independent on the size of the database, only dependent on the profile hmm and the target sequence.

## Data Availability

Data generated or analyzed during this study are included in this published article and its supplementary information files. Sequences used in this study are available on Genbank (NCBI) accession numbers OL334941 to OL334977.

## Supplementary Materials

The following supporting information can be downloaded at: https://www.mdpi.com/article/10.3390/microorganisms10051061/s1, Figure S1: strategy flow diagram for the identification and characterization of polygalacturonases in *Phytophthora cinnamomi*; Figure S2: pairwise alignment (zoomed out) of intron-containing PG genomic sequences with their corresponding coding sequences; Figure S3: pairwise alignment (zoomed in) of intron-containing PG genomic sequences with their corresponding coding sequences; Figure S4: a multiple sequence alignment of the polygalacturonase protein sequences from *Phytophthora cinnamomi*; Table S1: polygalacturonase sequences from different *Phytophthora* spp. used for the multiple sequence alignment; Table S2: polygalacturonase sequences from outgroup species and their ID as depicted on the phylogenetic trees; Table S3: polygalacturonase sequences from oomycete species and their ID as depicted on the phylogenetic tree; Table S4: representation of the intron exon boundaries of polygalacturonase genes from *Phytophthora cinnamomi*-GKB4 genome (accession: JAFJYM000000000) using Augustus for gene prediction; Table S5: protein sequence similarity of full-length and partial putative polygalacturonase proteins of *Phytophthora cinnamomi* to PG proteins from *Phytophthora* spp. based on BLASTP analyses; Table S6: full-length and partial polygalacturonase sequences from *Phytophthora cinnamomic*; Table S7: log 2-Fold Change gene expression of PG genes from *P. cinnamomi* at 12 hpi, 24 hpi and 120 hpi during colonization of the susceptible avocado rootstock (R. 012) normalized to mycelia. References [15,23,71,72] cited in Supplementary Materials.
