# Supplementary material for: The Identification and Characterization of Endopolygalacturonases in a South African Isolate of Phytophthora cinnamomi
Source: Microorganisms. 2022 May 20;10(5):1061. doi: 10.3390/microorganisms10051061 (PMC9146145; doi:10.3390/microorganisms10051061)
Supplement: Supplementary file 1 [file microorganisms-10-01061-s001.zip › microorganisms-1708351-supplementary.pdf]

The identification and characterization of endopolygalacturonases in a South African isolate of *Phytophthora cinnamomi*

Journal: Microorganisms

Authors: Tsakani M. Miyambo, Robert Backer, Fourie Joubert, Juanita Engelbrecht, Nicolaas A. van der Merwe, Noëlani van den Berg

*Address Correspondence to Tsakani M. Miyambo, tsakane.miyambo@fabi.up.ac.za

Affiliation: Department of Biochemistry, Genetics and Microbiology, University of Pretoria, Pretoria, Gauteng, South Africa.

Forestry and Agricultural Biotechnology Institute, University of Pretoria, Pretoria,

Table S1. Polygalacturonase sequences from different *Phytophthora* spp. used for the multiple sequence alignment.

Name	Species	Protein ID	Database	Full-length/Partial
PCIPG2	*Phytophthora capsici*	ABD72596	NCBI	Full-length
PCIPG5	*P. capsici*	ABU46393	NCBI	Full-length
PCIPG8	*P. capsici*	ADN18844	NCBI	Full-length
PCIPG9	*P. capsici*	ADN18850	NCBI	Full-length
PCIPG10	*P. capsici*	ADN18854	NCBI	Full-length
PCIPG11	*P. capsici*	ADN18855	NCBI	Full-length
PCIPG12	*P. capsici*	ADN18856	NCBI	Full-length
PCIPG13	*P. capsici*	ADN18842	NCBI	Full-length
PCIPG14	*P. capsici*	ADN18847	NCBI	Full-length
PCIPG15	*P. capsici*	ADN18851	NCBI	Full-length
PCIPG16	*P. capsici*	ADN18852	NCBI	Full-length
PCIPG17	*P. capsici*	ADN18853	NCBI	Full-length
PCIPG18	*P. capsici*	ADN18846	NCBI	Full-length
PCIPG19	*P. capsici*	ADN18849	NCBI	Partial
PCIPG20	*P. capsici*	ADN18845	NCBI	Full-length
PCIPG21	*P. capsici*	ADN18843	NCBI	Full-length
PCIPG22	*P. capsici*	ADN18848	NCBI	Full-length
PCPG1	*Phytophthora cinnamomi*	AAN34612	NCBI	Full-length
PCPG2	*P. cinnamomi*	AAN34613	NCBI	Full-length
PCPG3	*P. cinnamomi*	AAN34614	NCBI	Full-length
PCPG4	*P. cinnamomi*	AAN34615	NCBI	Full-length
PCPG6	*P. cinnamomi*	AAN34616	NCBI	Full-length
PCPG7	*P. cinnamomi*	AAN05466	NCBI	Full-length
PCPG8	*P. cinnamomi*	AAN05467	NCBI	Full-length
PCPG9	*P. cinnamomi*	AAN05468	NCBI	Full-length
PCPG10	*P. cinnamomi*	AAN05465	NCBI	Full-length
PCPG12	*P. cinnamomi*	AAN05460	NCBI	Full-length
PCPG14	*P. cinnamomi*	AAN05461	NCBI	Full-length
PCPG15	*P. cinnamomi*	AAN05462	NCBI	Full-length
PCPG16	*P. cinnamomi*	AAN05463	NCBI	Full-length

Table S1. Polygalacturonase sequences from different *Phytophthora spp.* used for the multiple sequence alignment.

Name	Species	Protein ID	Database	Full-length/Partial
PCPG19	*P. cinnamomi*	AAN05458	NCBI	Full-length
PIPG1	*P. infestans*	AAK98644	NCBI	Full-length
PIPG1-T40	*Phytophthora infestans*	XP_002996850	NCBI	Partial
PIPG2-T40	*P. infestans*	XP_002902085	NCBI	Full-length
PIPG3-T40	*P. infestans*	XP_002897269	NCBI	Full-length
PIPG4-T40	*P. infestans*	XP_002901692	NCBI	Full-length
PIPG5-T40	*P. infestans*	XP_002897268	NCBI	Full-length
PIPG6-T40	*P. infestans*	XP_002897267	NCBI	Full-length
PIPG7-T40	*P. infestans*	XP_002996983	NCBI	Full-length
PIPG8-T40	*P. infestans*	XP_002896211	NCBI	Partial
PIPG9-T40	*P. infestans*	XP_002896198	NCBI	Partial
PIPG10-T40	*P. infestans*	XP_002896197	NCBI	Partial
PIPG11-T40	*P. infestans*	XP_002896196	NCBI	Partial
PIPG12-T40	*P. infestans*	XP_002896192	NCBI	Partial
PIPG13-T40	*P. infestans*	XP_002896191	NCBI	Full-length
PIPG14-T40	*P. infestans*	XP_002896190	NCBI	Full-length
PIPG15-T40	*P. infestans*	XP_002896187	NCBI	Full-length
PIPG16-T40	*P. infestans*	XP_002896186	NCBI	Partial
PIPG17-T40	*P. infestans*	XP_002895235	NCBI	Partial
PIPG18-T40	*P. infestans*	XP_002895234	NCBI	Full-length
PIPG19-T40	*P. infestans*	XP_002895233	NCBI	Full-length
PPPG1	*Phytophthora parasitica*	AAW22993	dbCAN	Full-length
PPPG2	*P. parasitica*	ABV82735	dbCAN	Full-length
PPPG3	*P. parasitica*	ABV82736	dbCAN	Full-length
PPPG4	*P. parasitica*	ABV82737	dbCAN	Full-length
PPPG5	*P. parasitica*	ABV82738	dbCAN	Full-length
PPPG6	*P. parasitica*	ABV82739	dbCAN	Full-length
PPPG7	*P. parasitica*	ABV82740	dbCAN	Full-length
PPPG8	*P. parasitica*	ABV82741	dbCAN	Full-length
PPPG9	*P. parasitica*	ABV82742	dbCAN	Full-length
PPPG10	*P. parasitica*	ABV82743	dbCAN	Full-length

Table S1. Polygalacturonase sequences from different *Phytophthora spp.* used for the multiple sequence alignment.

Name	Species	Protein ID	Database	Full-length/Partial
PRPG1	*Phytophthora ramorum*	41820	JGI	Full-length
PRPG2	*P. ramorum*	72130	JGI	Partial
PRPG3	*P. ramorum*	40980	JGI	Full-length
PRPG4	*P. ramorum*	48642	JGI	Partial
PRPG5	*P. ramorum*	54493	JGI	Full-length
PRPG6	*P. ramorum*	54527	JGI	Full-length
PRPG7	*P. ramorum*	72133	JGI	Full-length
PRPG9	*P. ramorum*	72135	JGI	Full-length
PRPG10	*P. ramorum*	72134	JGI	Full-length
PRPG11	*P. ramorum*	83562	JGI	Full-length
PRPG13	*P. ramorum*	83564	JGI	Full-length
PRPG14	*P. ramorum*	83568	JGI	Full-length
PRPG15	*P. ramorum*	83573	JGI	Full-length
PRPG16	*P. ramorum*	83574	JGI	Full-length
PSPG1	*Phytophthora sojae*	247698	JGI	Full-length
PSPG2	*P. sojae*	250266	JGI	Full-length
PSPG3	*P. sojae*	259959	JGI	Full-length
PSPG4	*P. sojae*	260041	JGI	Full-length
PSPG5	*P. sojae*	260065	JGI	Full-length
PSPG6	*P. sojae*	322946	JGI	Full-length
PSPG7	*P. sojae*	487620	JGI	Partial
PSPG8	*P. sojae*	505673	JGI	Partial
PSPG12	*P. sojae*	522084	JGI	Full-length
PSPG13	*P. sojae*	259928	JGI	Full-length
PSPG14	*P. sojae*	259936	JGI	Full-length
PSPG15	*P. sojae*	259951	JGI	Full-length
PSPG16	*P. sojae*	333550	JGI	Full-length
PSPG17	*P. sojae*	333549	JGI	Full-length
PSPG18	*P. sojae*	430630	JGI	Full-length
PSPG19	*P. sojae*	505361	JGI	Full-length
PSPG20	*P. sojae*	506837	JGI	Full-length

Table S1. Polygalacturonase sequences from different *Phytophthora spp.* used for the multiple sequence alignment.

Name	Species	Protein ID	Database	Full-length/Partial
PSPG21	*P. sojae*	507865	JGI	Full-length
PSPG22	*P. sojae*	508703	JGI	Partial
PSPG23	*P. sojae*	509650	JGI	Partial
PSPG24	*P. sojae*	509900	JGI	Partial
PSPG25	*P. sojae*	330711	JGI	Partial

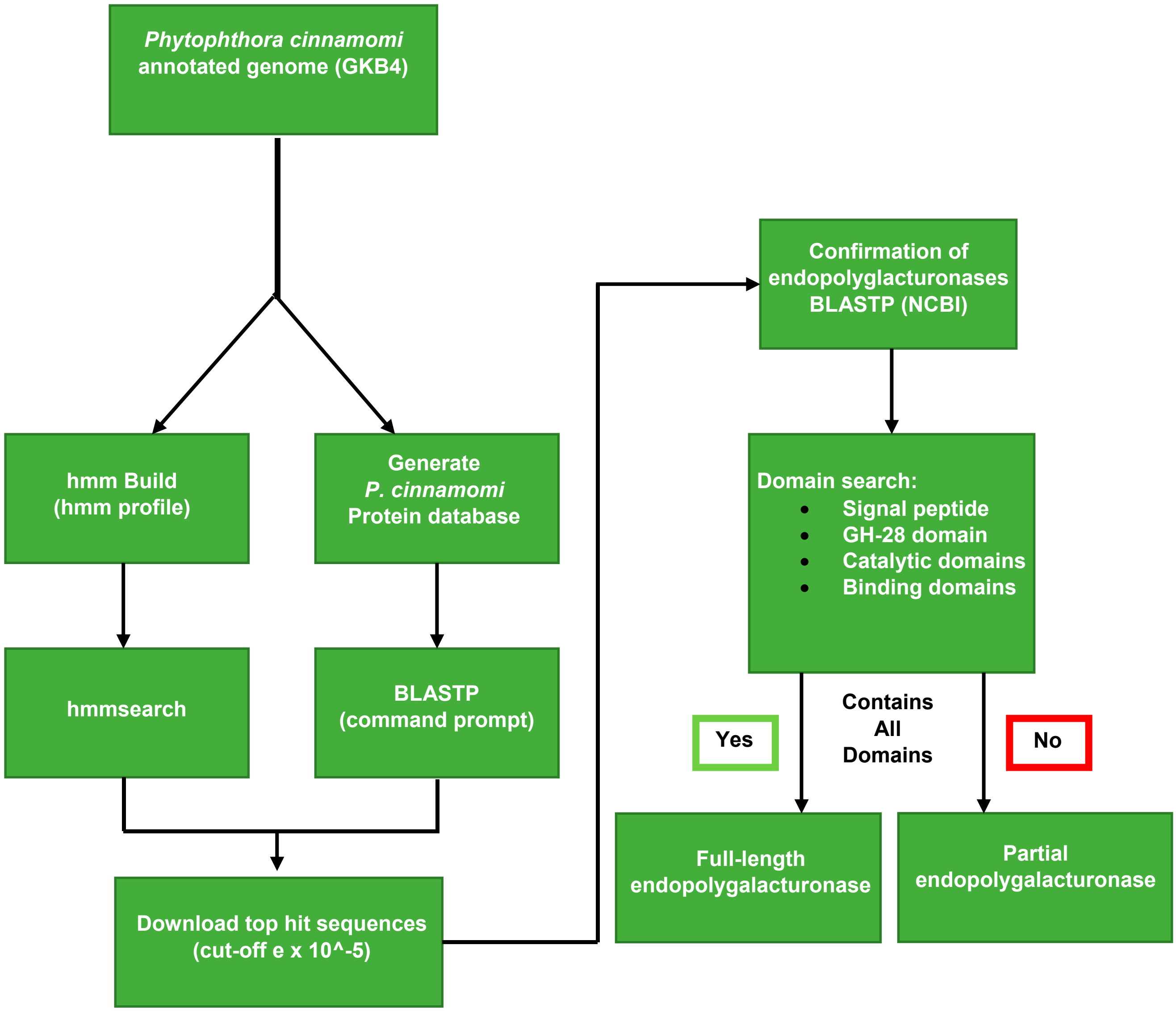

Figure S1. Strategy flow diagram for the identification and characterization of polygalacturonases in *Phytophthora cinnamomi*. A command line (hmm build) was used and run on a linux server to build an hmm profile from a multiple sequence alignment containing PG sequences from other *Phytophthora spp.* BLASTP analyses were also conducted using the server. The resultant sequences with a cut-off value ≤ E-value x10^{-5} were selected and BLASTP analyses were performed against the (NCBI-nr) to confirm that they were PG sequences. The sequences were subjected to signature domain search and classified as either full-length or partial sequences. The sequences that lacked either one or more domains were considered as partial sequence.

Table S2. Polygalacturonase sequences from outgroup species and their ID as depicted on the phylogenetic trees.

Outgroup Species	Tree ID	Accession number/ Protein ID	Trophic lifestyle	Signal peptide	Domain	Source
***Phytophthora cinnamomi* GKB4 Phylogenetic Tree**						
Pythium vexans	Py_vex_PG1_H	EPrPVT00000022936	Hemibiotrophic	1-18	I, II, III, IV & V	ENSEMBL PROTISTS
Pythium arrhenomanes	Py_arr_PG1_N	EPrPRT00000024470	Necrotrophic	1-22	I, II, III, IV & V	ENSEMBL PROTISTS
Pythium aphanidermatum	Py_aph_PG1_N	EPrPA00000023057	Necrotrophic	1-20	I, II, III, IV & V	ENSEMBL PROTISTS
Plasmopara halstedii	Pl_hal_PG1_B	CEG40133	Biotrophic	1-25	I, II, III, IV & V	NCBI
P. halstedii	Pl_hal_PG2_B	CEG49916	Biotrophic	1-20	I, II, III, IV & V	NCBI
Saprolegnia diclina	Sa_dic_PG1_N	XM_008613903	Necrotrophic	1-20	I, II, III, IV & V	NCBI
S. diclina	Sa_dic_PG2_N	XM_008605568	Necrotrophic	1-20	I, II, III, IV & V	NCBI
S. diclina	Sa_dic_PG3_N	XP_008612126	Necrotrophic	1-20	I, II, III, IV & V	NCBI
**Oomycetes Phylogenetic Tree**						
Fragilariopsis cylindrus	Fl_cyl_PG1_Outgroup	KV784353	---	1-28	I & II	NCBI

Table S3. Polygalacturonase sequences from oomycete species and their ID as depicted on the phylogenetic tree.

Species	Tree ID	Accession number/ Protein ID	Trophic lifestyle	Signal peptide	Domain	Source
Phytophthora cinnamomi	Ph_cin_PG1_H	AAN34612/ 243190	Hemibiotrophic	1-20	I, II, III, IV & V	NCBI/ JGI
P. cinnamomi	Ph_cin_PG2_H	AAN34613	Hemibiotrophic	1-20	I, II, III, IV & V	NCBI
P. cinnamomi	Ph_cin_PG3_H	AAN34614	Hemibiotrophic	1-20	I, II, III, IV & V	NCBI
P. cinnamomi	Ph_cin_PG4_H	AAN34615	Hemibiotrophic	1-20	I, II, III, IV & V	NCBI
P. cinnamomi	Ph_cin_PG5_H	94850	Hemibiotrophic	1-19	I, II, III, IV & V	JGI
P. cinnamomi	Ph_cin_PG6_H	AAN34616	Hemibiotrophic	1-20	I, II, III, IV & V	NCBI
P. cinnamomi	Ph_cin_PG7_H	AF398946	Hemibiotrophic	1-20	I, II, III, IV & V	NCBI
P. cinnamomi	Ph_cin_PG8_H	AF398947	Hemibiotrophic	1-20	I, II, III, IV & V	NCBI
P. cinnamomi	Ph_cin_PG9_H	AF398948	Hemibiotrophic	1-20	I, II, III, IV & V	NCBI
P. cinnamomi	Ph_cin_PG10_H	AF398945	Hemibiotrophic	1-20	I, II, III, IV & V	NCBI
P. cinnamomi	Ph_cin_PG11_H	243146	Hemibiotrophic	1-20	I, II, III, IV & V	JGI
P. cinnamomi	Ph_cin_PG12_H	AF398938	Hemibiotrophic	1-22	I, II, III, IV & V	NCBI
P. cinnamomi	Ph_cin_PG13_H	243199	Hemibiotrophic	1-20	I, II, III, IV & V	JGI
P. cinnamomi	Ph_cin_PG14_H	AF398940	Hemibiotrophic	1-22	I, II, III, IV & V	NCBI
P. cinnamomi	Ph_cin_PG15_H	AF398941	Hemibiotrophic	1-22	I, III, IV & V	NCBI
P. cinnamomi	Ph_cin_PG16_H	AF398942/ 243454	Hemibiotrophic	1-22	I, II, III, IV & V	NCBI/ JGI
P. cinnamomi	Ph_cin_PG17_H	243222	Hemibiotrophic	1-20	I, II, III, IV & V	JGI

Table S3. Polygalacturonase sequences from oomycete species and their ID as depicted on the phylogenetic tree.

Species	Tree ID	Accession number/ Protein ID	Trophic lifestyle	Signal peptide	Domain	Source
P. cinnamomi	Ph_cin_PG18_H	321736	Hemibiotrophic	1-20	I, II, III, IV & V	JGI
P. cinnamomi	Ph_cin_PG19_H	AAN24612	Hemibiotrophic	1-20	I, II, III, IV & V	NCBI
P. cinnamomi	Ph_cin_PG20_H	557459	Hemibiotrophic	1-20	I, II, III, IV & V	JGI
P. cinnamomi	Ph_cin_PG21_H	557452	Hemibiotrophic	1-20	I, II, III, IV & V	JGI
P. cinnamomi	Ph_cin_PG22_H	557447	Hemibiotrophic	1-20	I, II, III, IV & V	JGI
P. cinnamomi	Ph_cin_PG23_H	14823	Hemibiotrophic	1-20	I, II, III, IV & V	JGI
P. cinnamomi	Ph_cin_PG24_H	258598	Hemibiotrophic	1-22	I, II, III, IV & V	JGI
P. cinnamomi	Ph_cin_PG25_H	270732	Hemibiotrophic	1-20	I, II, III, IV & V	JGI
P. cinnamomi	PcPG1	OL334941	Hemibiotrophic	1-20	I, II, III, IV & V	ARP, Unpublished data
P. cinnamomi	PcPG2	OL334942	Hemibiotrophic	1-20	I, II, III, IV & V	ARP, Unpublished data
P. cinnamomi	PcPG3	OL334943	Hemibiotrophic	1-20	I, II, III, IV & V	ARP, Unpublished data
P. cinnamomi	PcPG4	OL334944	Hemibiotrophic	1-20	I, II, III, IV & V	ARP, Unpublished data
P. cinnamomi	PcPG5	OL334945	Hemibiotrophic	1-20	I, II, III, IV & V	ARP, Unpublished data
P. cinnamomi	PcPG6	OL334946	Hemibiotrophic	1-20	I, II, III, IV & V	ARP, Unpublished data
P. cinnamomi	PcPG7	OL334947	Hemibiotrophic	1-20	I, II, III, IV & V	ARP, Unpublished data
P. cinnamomi	PcPG8	OL334948	Hemibiotrophic	1-20	I, II, III, IV & V	ARP, Unpublished data
P. cinnamomi	PcPG9	OL334949	Hemibiotrophic	1-20	I, II, III, IV & V	ARP, Unpublished data
P. cinnamomi	PcPG10	OL334950	Hemibiotrophic	1-20	I, II, III, IV & V	ARP, Unpublished data

Table S3. Polygalacturonase sequences from oomycete species and their ID as depicted on the phylogenetic tree.						
**Species**	**Tree ID**	**Accession number/ Protein ID**	**Trophic lifestyle**	**Signal peptide**	**Domain**	**Source**
P. cinnamomi	PcPG11	OL334951	Hemibiotrophic	1-20	I, II, III, IV & V	ARP, Unpublished data
P. cinnamomi	PcPG12	OL334952	Hemibiotrophic	1-20	I, II, III, IV & V	ARP, Unpublished data
P. cinnamomi	PcPG13	OL334953	Hemibiotrophic	1-20	I, II, III, IV & V	ARP, Unpublished data
P. cinnamomi	PcPG14	OL334954	Hemibiotrophic	1-20	I, II, III, IV & V	ARP, Unpublished data
P. cinnamomi	PcPG16	OL334956	Hemibiotrophic	1-20	I, II, III, IV & V	ARP, Unpublished data
P. cinnamomi	PcPG17	OL334957	Hemibiotrophic	1-20	I, II, III, IV & V	ARP, Unpublished data
P. cinnamomi	PcPG19	OL334959	Hemibiotrophic	1-22	I, II, III, IV & V	ARP, Unpublished data
P. cinnamomi	PcPG22	OL334962	Hemibiotrophic	1-19	I, II, III, IV & V	ARP, Unpublished data
P. cinnamomi	PcPG25	OL334965	Hemibiotrophic	1-22	I, II, III, IV & V	ARP, Unpublished data
Phytophthora capsici	Ph_cap_PG1_H	DQ415987	Hemibiotrophic	1-19	I, II, III, IV & V	NCBI
P. capsici	Ph_cap_PG2_H	EF558847	Hemibiotrophic	1-20	I, II, III, IV & V	NCBI
P. capsici	Ph_cap_PG3_H	GQ856521	Hemibiotrophic	1-20	I, II, III, IV & V	NCBI
P. capsici	Ph_cap_PG4_H	GQ856527	Hemibiotrophic	1-20	I, II, III, IV & V	NCBI
P. capsici	Ph_cap_PG5_H	GQ856531	Hemibiotrophic	1-20	I, II, III, IV & V	NCBI
P. capsici	Ph_cap_PG6_H	GQ856532	Hemibiotrophic	1-20	I, II, III, IV & V	NCBI
P. capsici	Ph_cap_PG7_H	GQ856533	Hemibiotrophic	1-19	I, II, III, IV & V	NCBI
P. capsici	Ph_cap_PG8_H	GQ856519	Hemibiotrophic	1-20	I, II, III, IV & V	NCBI

Table S3. Polygalacturonase sequences from oomycete species and their ID as depicted on the phylogenetic tree.

Species	Tree ID	Accession number/ Protein ID	Trophic lifestyle	Signal peptide	Domain	Source
P. capsici	Ph_cap_PG9_H	GQ856524	Hemibiotrophic	1-20	I, II, III, IV & V	NCBI
P. capsici	Ph_cap_PG10_H	GQ856528	Hemibiotrophic	1-20	I, II, III, IV & V	NCBI
P. capsici	Ph_cap_PG11_H	GQ856529	Hemibiotrophic	1-20	I, II, III, IV & V	NCBI
P. capsici	Ph_cap_PG12_H	GQ856530	Hemibiotrophic	1-20	I, II, III, IV & V	NCBI
P. capsici	Ph_cap_PG13_H	GQ856523	Hemibiotrophic	1-20	I, II, III, IV & V	NCBI
P. capsici	Ph_cap_PG14_H	GQ856526	Hemibiotrophic	1-19	I, II, III, IV & V	NCBI
P. capsici	Ph_cap_PG15_H	GQ856522	Hemibiotrophic	1-19	I, II, III, IV & V	NCBI
P. capsici	Ph_cap_PG16_H	GQ856520	Hemibiotrophic	1-19	I, II, III, IV & V	NCBI
P. capsici	Ph_cap_PG17_H	GQ856525	Hemibiotrophic	1-22	I, II, III, IV & V	NCBI
Phytophthora infestans	Ph_inf_PG1_H	AAK98644	Hemibiotrophic	1-20	I, II, III & IV	NCBI
P. infestans	Ph_inf_PG2_H	XM_002902039	Hemibiotrophic	1-20	I, II, III, IV & V	NCBI
P. infestans	Ph_inf_PG3_H	XM_002897222	Hemibiotrophic	1-20	I, II, III, IV & V	NCBI
P. infestans	Ph_inf_PG4_H	XM_002896144	Hemibiotrophic	1-20	I, II, III, IV & V	NCBI
P. infestans	Ph_inf_PG5_H	XM_002896141	Hemibiotrophic	1-20	I, II, III, IV & V	NCBI
P. infestans	Ph_inf_PG6_H	XM_002895188	Hemibiotrophic	1-20	I, II, III, IV & V	NCBI
P. infestans	Ph_inf_PG7_H	XM_002895187	Hemibiotrophic	1-20	I, II, III, IV & V	NCBI
P. infestans	Ph_inf_PG8_H	XM_002896145	Hemibiotrophic	1-20	I, II, III, IV & V	NCBI

Table S3. Polygalacturonase sequences from oomycete species and their ID as depicted on the phylogenetic tree.

Species	Tree ID	Accession number/ Protein ID	Trophic lifestyle	Signal peptide	Domain	Source
P. infestans	Ph_inf_PG9_H	XM_002901646	Hemibiotrophic	1-19	I, II, III, IV & V	NCBI
Phytophthora parasitica	Ph_par_PG1_H	AAW22993	Hemibiotrophic	1-20	I, II, IV & V	NCBI
P. parasitica	Ph_par_PG2_H	ABV82735	Hemibiotrophic	1-20	I, II, III, IV & V	NCBI
P. parasitica	Ph_par_PG3_H	ABV82736	Hemibiotrophic	1-20	I, II, III, IV & V	NCBI
P. parasitica	Ph_par_PG4_H	ABV82737	Hemibiotrophic	1-20	I, II, III, IV & V	NCBI
P. parasitica	Ph_par_PG5_H	ABV82738	Hemibiotrophic	1-19	I, II, III, IV & V	NCBI
P. parasitica	Ph_par_PG6_H	ABV82739	Hemibiotrophic	1-20	I, II, III, IV & V	NCBI
P. parasitica	Ph_par_PG7_H	ABV82740	Hemibiotrophic	1-17	I, II, III, IV & V	NCBI
P. parasitica	Ph_par_PG8_H	ABV82741	Hemibiotrophic	1-20	I, II, III, IV & V	NCBI
P. parasitica	Ph_par_PG9_H	ABV82742	Hemibiotrophic	1-19	I, II, III, IV & V	NCBI
P. parasitica	Ph_par_PG10_H	ABV82743	Hemibiotrophic	1-20	I, II, III, IV & V	NCBI
Phytophthora ramorum	Ph_ram_PG1_H	72135	Hemibiotrophic	1-20	I, II, III, IV & V	JGI
P. ramorum	Ph_ram_PG2_H	83562	Hemibiotrophic	1-20	I, II, III, IV & V	JGI
P. ramorum	Ph_ram_PG3_H	72134	Hemibiotrophic	1-20	I, II, III, IV & V	JGI
P. ramorum	Ph_ram_PG4_H	83565	Hemibiotrophic	1-20	I, II, III, IV & V	JGI
P. ramorum	Ph_ram_PG5_H	83564	Hemibiotrophic	1-20	I, II, III, IV & V	JGI
P. ramorum	Ph_ram_PG6_H	83568	Hemibiotrophic	1-20	I, II, III, IV & V	JGI

Table S3. Polygalacturonase sequences from oomycete species and their ID as depicted on the phylogenetic tree.

Species	Tree ID	Accession number/ Protein ID	Trophic lifestyle	Signal peptide	Domain	Source
P. ramorum	Ph_ram_PG9_H	83574	Hemibiotrophic	1-20	I, II, III, IV & V	JGI
Phytophthora sojae	Ph_soj_PG1_H	260065	Hemibiotrophic	1-20	I, II, III & V	JGI
P. sojae	Ph_soj_PG2_H	260041	Hemibiotrophic	1-20	I, II, III, IV & V	JGI
P. sojae	Ph_soj_PG3_H	259936	Hemibiotrophic	1-20	I, II, III, IV & V	JGI
P. sojae	Ph_soj_PG4_H	259959	Hemibiotrophic	1-19	I, II, III, IV & V	JGI
P. sojae	Ph_soj_PG5_H	250266	Hemibiotrophic	1-19	I, II, III, IV & V	JGI
P. sojae	Ph_soj_PG6_H	322946	Hemibiotrophic	1-22	I, II, III, IV & V	JGI
P. sojae	Ph_soj_PG7_H	507025	Hemibiotrophic	1-20	I, II, III, IV & V	JGI
P. sojae	Ph_soj_PG8_H	506837	Hemibiotrophic	1-20	I, II, III, IV & V	JGI
P. sojae	Ph_soj_PG9_H	507865	Hemibiotrophic	1-20	I, II, III, IV & V	JGI
P. sojae	Ph_soj_PG10_H	508773	Hemibiotrophic	1-20	I, II, III, IV & V	JGI
P. sojae	Ph_soj_PG11_H	508703	Hemibiotrophic	1-19	I, II, III, IV & V	JGI
P. sojae	Ph_soj_PG12_H	509867	Hemibiotrophic	1-20	I, II, III, IV & V	JGI
P. sojae	Ph_soj_PG13_H	509900	Hemibiotrophic	1-20	I, II, III, IV & V	JGI
P. sojae	Ph_soj_PG14_H	509650	Hemibiotrophic	1-20	I, II, III, IV & V	JGI
P. sojae	Ph_soj_PG15_H	505673	Hemibiotrophic	1-20	I, II, III, IV & V	JGI
P. sojae	Ph_soj_PG16_H	259928	Hemibiotrophic	1-20	I, II, III, IV & V	JGI

Table S3. Polygalacturonase sequences from oomycete species and their ID as depicted on the phylogenetic tree.

Species	Tree ID	Accession number/ Protein ID	Trophic lifestyle	Signal peptide	Domain	Source
Phytophthora megakarya	Ph_meg_PG1_H	OWY94663	Hemibiotrophic	1-20	I, II, III, IV & V	NCBI
P. megakarya	Ph_meg_PG2_H	OWY98164	Hemibiotrophic	1-20	I, II, III, IV & V	NCBI
P. megakarya	Ph_meg_PG3_H	OWZ12176	Hemibiotrophic	1-20	I, II, III, IV & V	NCBI
P. megakarya	Ph_meg_PG4_H	OWY97638	Hemibiotrophic	1-20	I, II, III, IV & V	NCBI
P. megakarya	Ph_meg_PG5_H	OWZ12175	Hemibiotrophic	1-20	I, II, III, IV & V	NCBI
P. megakarya	Ph_meg_PG6_H	OWZ12177	Hemibiotrophic	1-20	I, II, III, IV & V	NCBI
P. megakarya	Ph_meg_PG7_H	OWY98906	Hemibiotrophic	1-20	I, II, III, IV & V	NCBI
Phytophthora nicotianae	Ph_nic_PG1_H	KUF86279	Hemibiotrophic	1-20	I, II, III, IV & V	NCBI
P. nicotianae	Ph_nic_PG2_H	KUF77588	Hemibiotrophic	1-20	I, II, III, IV & V	NCBI
P. nicotianae	Ph_nic_PG3_H	KUF94022	Hemibiotrophic	1-20	I, II, III, IV & V	NCBI
P. nicotianae	Ph_nic_PG4_H	KUF90172	Hemibiotrophic	1-19	I, II, IV & V	NCBI
P. nicotianae	Ph_nic_PG5_H	KUF84330	Hemibiotrophic	1-20	I, II, III, IV & V	NCBI
P. nicotianae	Ph_nic_PG6_H	KUF94598	Hemibiotrophic	1-20	I, II, III, IV & V	NCBI
P. nicotianae	Ph_nic_PG7_H	KUF94020	Hemibiotrophic	1-20	I, II, III, IV & V	NCBI
P. nicotianae	Ph_nic_PG8_H	KUF94603	Hemibiotrophic	1-20	I, II, III, IV & V	NCBI
P. nicotianae	Ph_nic_PG9_H	KUF91066	Hemibiotrophic	1-20	I, II, III, IV & V	NCBI
P. nicotianae	Ph_nic_PG10_H	KUF91067	Hemibiotrophic	1-20	I, II, III, IV & V	NCBI

Table S3. Polygalacturonase sequences from oomycete species and their ID as depicted on the phylogenetic tree.

Species	Tree ID	Accession number/ Protein ID	Trophic lifestyle	Signal peptide	Domain	Source
P. nicotianae	Ph_nic_PG11_H	KUF94602	Hemibiotrophic	1-17	I, II, III, IV & V	NCBI
P. nicotianae	Ph_nic_PG12_H	KUF78020	Hemibiotrophic	1-19	I, II, III, IV & V	NCBI
P. nicotianae	Ph_nic_PG13_H	KUF84327	Hemibiotrophic	1-20	I, II, III, IV & V	NCBI
P. nicotianae	Ph_nic_PG14_H	KUF94019	Hemibiotrophic	1-20	I, II, III, IV & V	NCBI
P. nicotianae	Ph_nic_PG15_H	KUF86106	Hemibiotrophic	1-20	I, II, III, IV & V	NCBI
Pythium vexans	Py_vex_PG1_H	EPrPVT00000022936	Hemibiotrophic	1-18	I, II, III, IV & V	ENSEMBL PROTISTS
Pythium arrhenomanes	Py_arr_PG1_N	EPrPRT00000024470	Necrotrophic	1-22	I, II, III, IV & V	ENSEMBL PROTISTS
Pythium aphanidermatum	Py_aph_PG1_N	EPrPA00000023057	Necrotrophic	1-20	I, II, III, IV & V	ENSEMBL PROTISTS
Plasmopara halstedii	Pl_hal_PG1_B	CEG40133	Biotrophic	1-25	I, II, III, IV & V	NCBI
P. halstedii	Pl_hal_PG2_B	CEG49916	Biotrophic	1-20	I, II, III, IV & V	NCBI

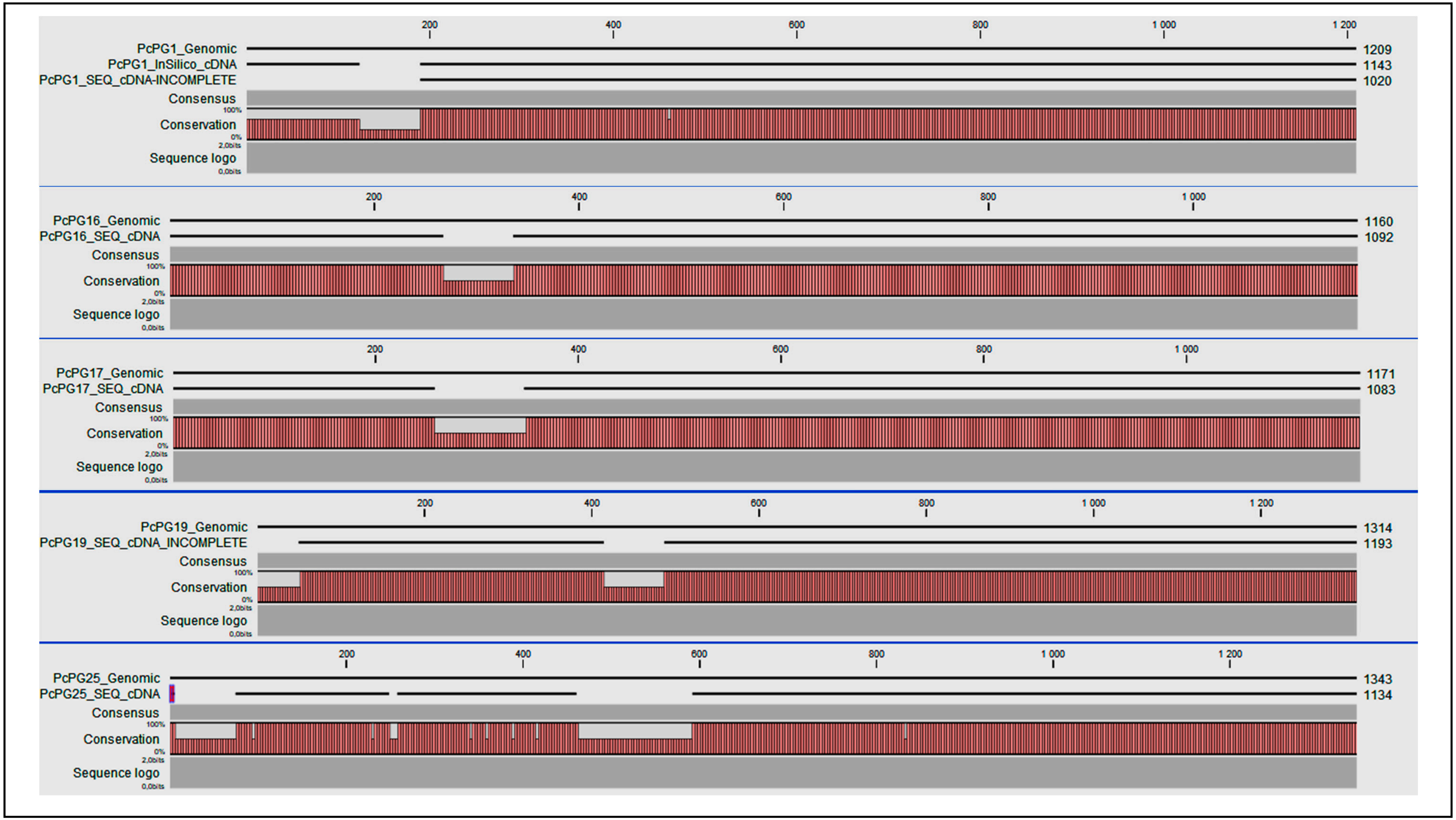
PcPG1_Genomic 1209
PcPG1_InSilico_cDNA 1143
PcPG1_SEQ_cDNA-INCOMPLETE 1020
Consensus
Conservation
Sequence logo
PcPG16_Genomic 1160
PcPG16_SEQ_cDNA 1092
PcPG17_Genomic 1171
PcPG17_SEQ_cDNA 1083
PcPG19_Genomic 1314
PcPG19_SEQ_cDNA_INCOMPLETE 1193
PcPG25_Genomic 1343
PcPG25_SEQ_cDNA 1134

Figure S2. Pairwise alignment (zoomed out) of intron-containing PG genomic sequences with their corresponding coding sequences. The genomic sequences of the introns are shown with a solid grey line, below are the coding sequences of the respective PG genes. One intron is present in each PG gene as shown by the gap in the coding sequence marked as the cDNA sequence. The gap in the N-terminal region of *pcpg19* and *pcpg25* is not an indication of an intron region, the consensus sequence could not be attained in these regions due to many conflicts observed in these nucleotides using CLC Main Workbench. The *In silico* identified coding sequence of *pcpg1* was included to show the intron position as we were unable to get the consensus sequence of the beginning of the sequence. We do not expect to see an intron in the N-terminal region, therefore we suggest that this gene also has one intron as predicted *in silico*. The consensus sequence is shown below the alignment with a grey solid rectangular block and the sequence conservation is marked in light and dark maroon.

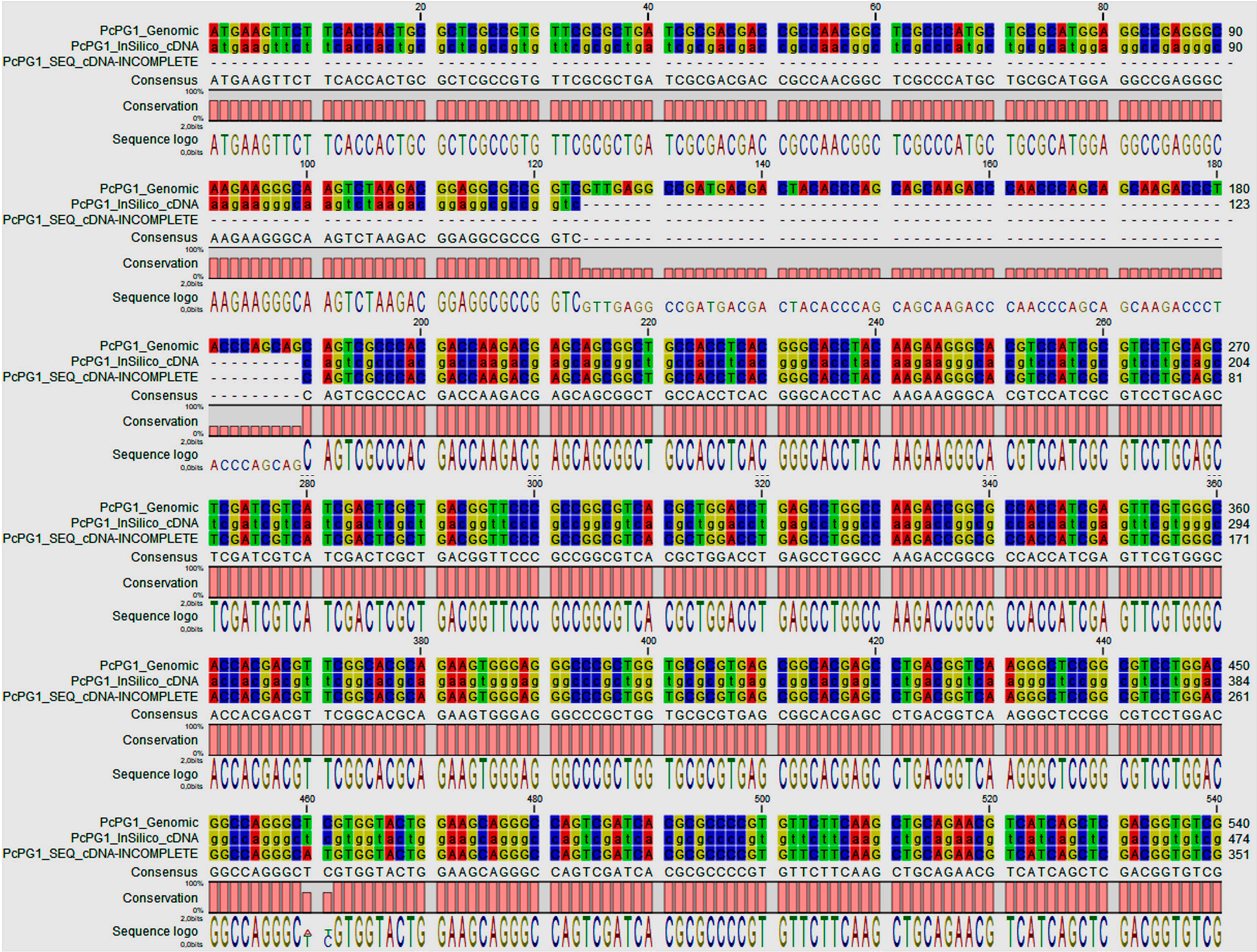
PcPG1_Genomic
PcPG1_InSilico_cDNA
PcPG1_SEQ_cDNA-INCOMPLETE
Consensus
Conservation
Sequence logo

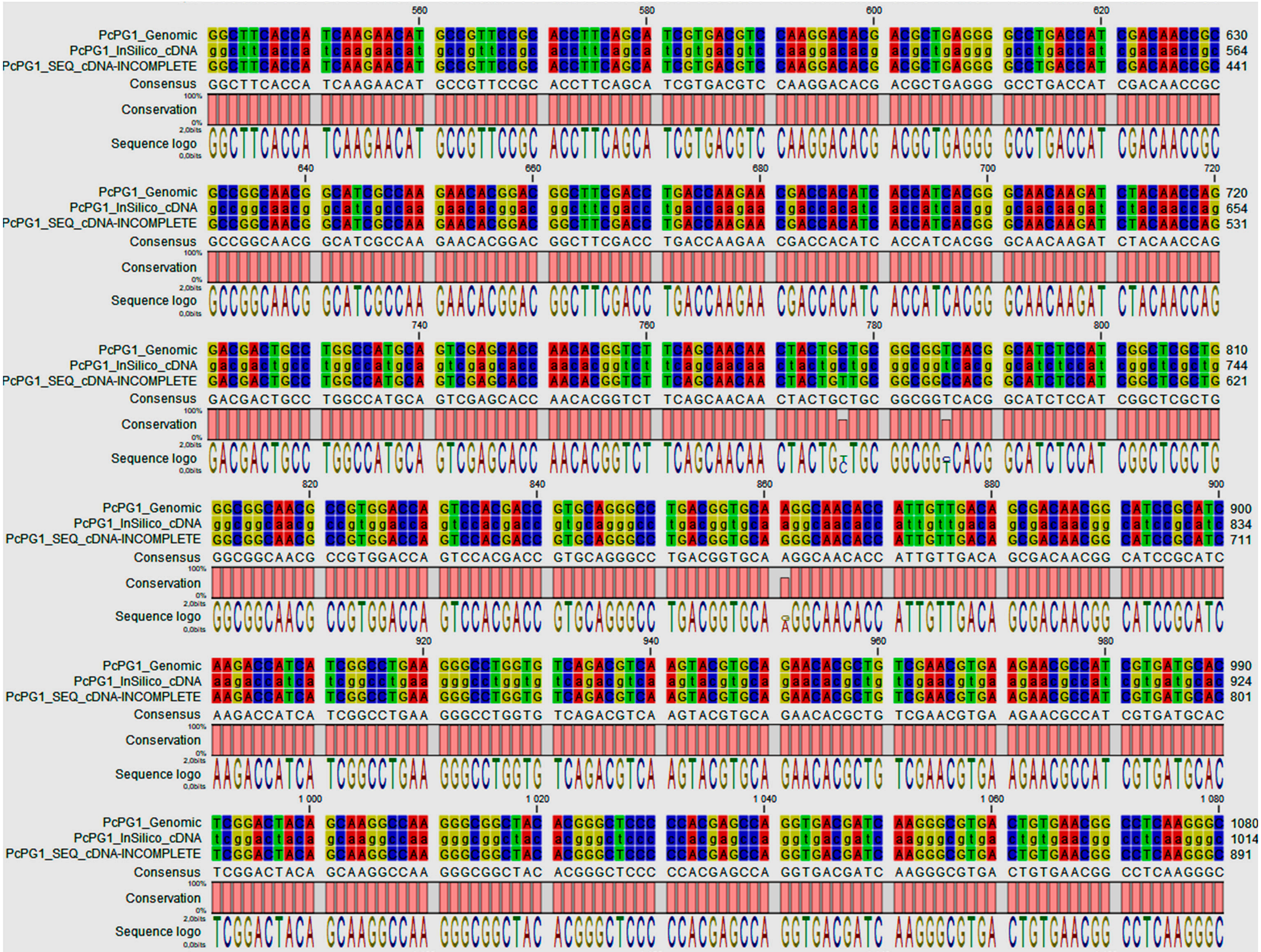

560 580 600 620
PcPG1_Genomic 630
PcPG1_InSilico_cDNA 564
PcPG1_SEQ_cDNA-INCOMPLETE 441
Consensus GGCTTCACCA TCAAGAACAT GCCGTTCCGC ACCTTCAGCA TCGTGACGTC CAAGGACACG ACGCTGAGGG GCCTGACCAT CGACAACCGC
Conservation
Sequence logo
640 660 680 700 720
PcPG1_Genomic 720
PcPG1_InSilico_cDNA 654
PcPG1_SEQ_cDNA-INCOMPLETE 531
Consensus GCCGGCAACG GCATCGCCAA GAACACGGAC GGCTTCGACC TGACCAAGAA CGACCACATC ACCATCACGG GCAACAAGAT CTACAACCAG
Conservation
Sequence logo
740 760 780 800
PcPG1_Genomic 810
PcPG1_InSilico_cDNA 744
PcPG1_SEQ_cDNA-INCOMPLETE 621
Consensus GACGACTGCC TGGCCATGCA GTCGAGCACC AACACGGTCT TCAGCAACAA CTACTGCTGC GGCGGTCACG GCATCTCCAT CGGCTCGCTG
Conservation
Sequence logo
820 840 860 880 900
PcPG1_Genomic 900
PcPG1_InSilico_cDNA 834
PcPG1_SEQ_cDNA-INCOMPLETE 711
Consensus GGCGGCAACG CCGTGGACCA GTCCACGACC GTGCAGGGCC TGACGGTGCA AGGCAACACC ATTGTTGACA GCGACAACGG CATCCGCATC
Conservation
Sequence logo
920 940 960 980
PcPG1_Genomic 990
PcPG1_InSilico_cDNA 924
PcPG1_SEQ_cDNA-INCOMPLETE 801
Consensus AAGACCATCA TCGGCCTGAA GGGCCTGGTG TCAGACGTCA AGTACGTGCA GAACACGCTG TCGAACGTGA AGAACGCCAT CGTGATGCAC
Conservation
Sequence logo
1 000 1 020 1 040 1 060 1 080
PcPG1_Genomic 1080
PcPG1_InSilico_cDNA 1014
PcPG1_SEQ_cDNA-INCOMPLETE 891
Consensus TCGGACTACA GCAAGGCCAA GGGCGGCTAC ACGGGCTCCC CCACGAGCCA GGTGACGATC AAGGGCGTGA CTGTGAACGG CCTCAAGGGC
Conservation
Sequence logo

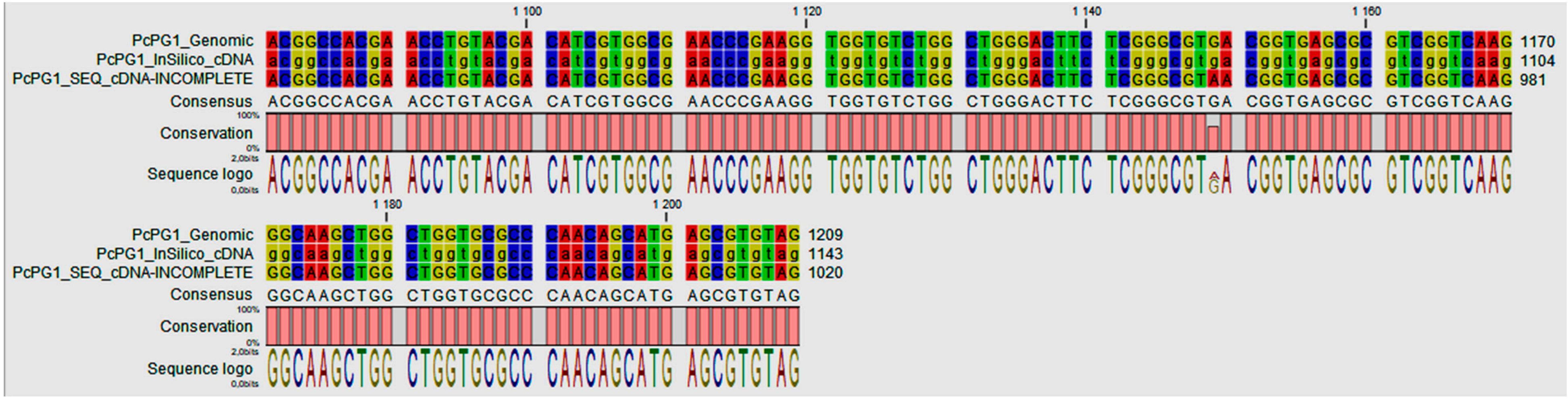
PcPG1_Genomic
PcPG1_InSilico_cDNA
PcPG1_SEQ_cDNA-INCOMPLETE
Consensus
Conservation
Sequence logo
1 100
1 120
1 140
1 160
1170
1104
981
ACGGCCACGA ACCTGTACGA CATCGTGGCG AACCCGAAGG TGGTGTCTGG CTGGGACTTC TCGGGCGTGA CGGTGAGCGC GTCGGTCAAG
1 180
1 200
1209
1143
1020
GGCAAGCTGG CTGGTGCGCC CAACAGCATG AGCGTGTAG

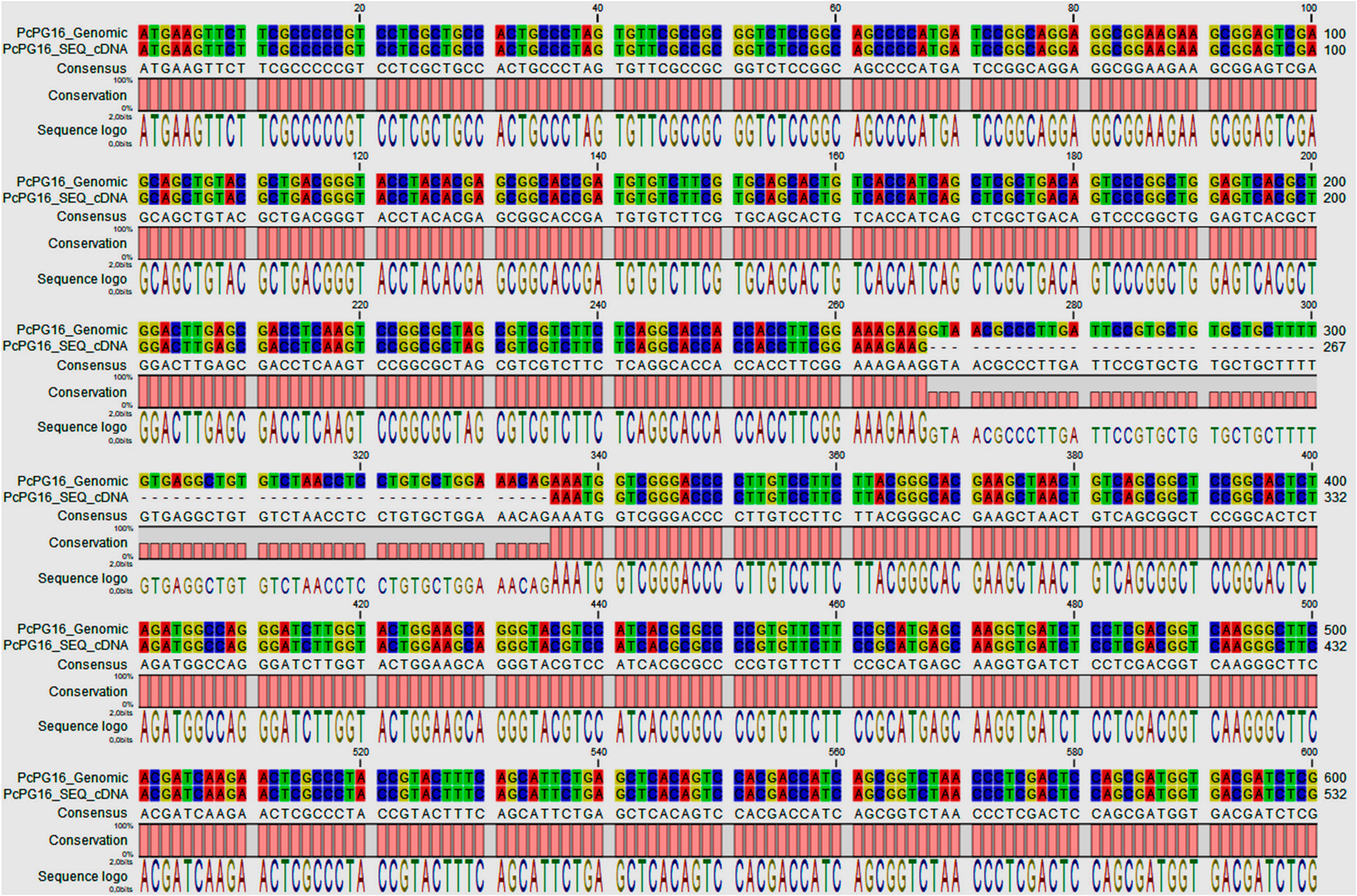
PcPG16_Genomic ATGAAGTTCT TCGCCCCCGT CCTCGCTGCC ACTGCCCTAG TGTTCGCCGC GGTCTCCGGC AGCCCCATGA TCCGGCAGGA GGCGGAAGAA GCGGAGTCGA 100
PcPG16_SEQ_cDNA ATGAAGTTCT TCGCCCCCGT CCTCGCTGCC ACTGCCCTAG TGTTCGCCGC GGTCTCCGGC AGCCCCATGA TCCGGCAGGA GGCGGAAGAA GCGGAGTCGA 100
Consensus ATGAAGTTCT TCGCCCCCGT CCTCGCTGCC ACTGCCCTAG TGTTCGCCGC GGTCTCCGGC AGCCCCATGA TCCGGCAGGA GGCGGAAGAA GCGGAGTCGA
Conservation
Sequence logo
PcPG16_Genomic GCAGCTGTAC GCTGACGGGT ACCTACACGA GCGGCACCGA TGTGTCTTCG TGCAGCACTG TCACCATCAG CTCGCTGACA GTCCCGGCTG GAGTCACGCT 200
PcPG16_SEQ_cDNA GCAGCTGTAC GCTGACGGGT ACCTACACGA GCGGCACCGA TGTGTCTTCG TGCAGCACTG TCACCATCAG CTCGCTGACA GTCCCGGCTG GAGTCACGCT 200
Consensus GCAGCTGTAC GCTGACGGGT ACCTACACGA GCGGCACCGA TGTGTCTTCG TGCAGCACTG TCACCATCAG CTCGCTGACA GTCCCGGCTG GAGTCACGCT
PcPG16_Genomic GGACTTGAGC GACCTCAAGT CCGGCGCTAG CGTCGTCTTC TCAGGCACCA CCACCTTCGG AAAGAAGGTA ACGCCCTTGA TTCCGTGCTG TGCTGCTTTT 300
PcPG16_SEQ_cDNA GGACTTGAGC GACCTCAAGT CCGGCGCTAG CGTCGTCTTC TCAGGCACCA CCACCTTCGG AAAGAAG--- ---------- ---------- ---------- 267
Consensus GGACTTGAGC GACCTCAAGT CCGGCGCTAG CGTCGTCTTC TCAGGCACCA CCACCTTCGG AAAGAAGGTA ACGCCCTTGA TTCCGTGCTG TGCTGCTTTT
PcPG16_Genomic GTGAGGCTGT GTCTAACCTC CTGTGCTGGA AACAGAAATG GTCGGGACCC CTTGTCCTTC TTACGGGCAC GAAGCTAACT GTCAGCGGCT CCGGCACTCT 400
PcPG16_SEQ_cDNA ---------- ---------- ---------- -----AAATG GTCGGGACCC CTTGTCCTTC TTACGGGCAC GAAGCTAACT GTCAGCGGCT CCGGCACTCT 332
Consensus GTGAGGCTGT GTCTAACCTC CTGTGCTGGA AACAGAAATG GTCGGGACCC CTTGTCCTTC TTACGGGCAC GAAGCTAACT GTCAGCGGCT CCGGCACTCT
PcPG16_Genomic AGATGGCCAG GGATCTTGGT ACTGGAAGCA GGGTACGTCC ATCACGCGCC CCGTGTTCTT CCGCATGAGC AAGGTGATCT CCTCGACGGT CAAGGGCTTC 500
PcPG16_SEQ_cDNA AGATGGCCAG GGATCTTGGT ACTGGAAGCA GGGTACGTCC ATCACGCGCC CCGTGTTCTT CCGCATGAGC AAGGTGATCT CCTCGACGGT CAAGGGCTTC 432
Consensus AGATGGCCAG GGATCTTGGT ACTGGAAGCA GGGTACGTCC ATCACGCGCC CCGTGTTCTT CCGCATGAGC AAGGTGATCT CCTCGACGGT CAAGGGCTTC
PcPG16_Genomic ACGATCAAGA ACTCGCCCTA CCGTACTTTC AGCATTCTGA GCTCACAGTC CACGACCATC AGCGGTCTAA CCCTCGACTC CAGCGATGGT GACGATCTCG 600
PcPG16_SEQ_cDNA ACGATCAAGA ACTCGCCCTA CCGTACTTTC AGCATTCTGA GCTCACAGTC CACGACCATC AGCGGTCTAA CCCTCGACTC CAGCGATGGT GACGATCTCG 532
Consensus ACGATCAAGA ACTCGCCCTA CCGTACTTTC AGCATTCTGA GCTCACAGTC CACGACCATC AGCGGTCTAA CCCTCGACTC CAGCGATGGT GACGATCTCG

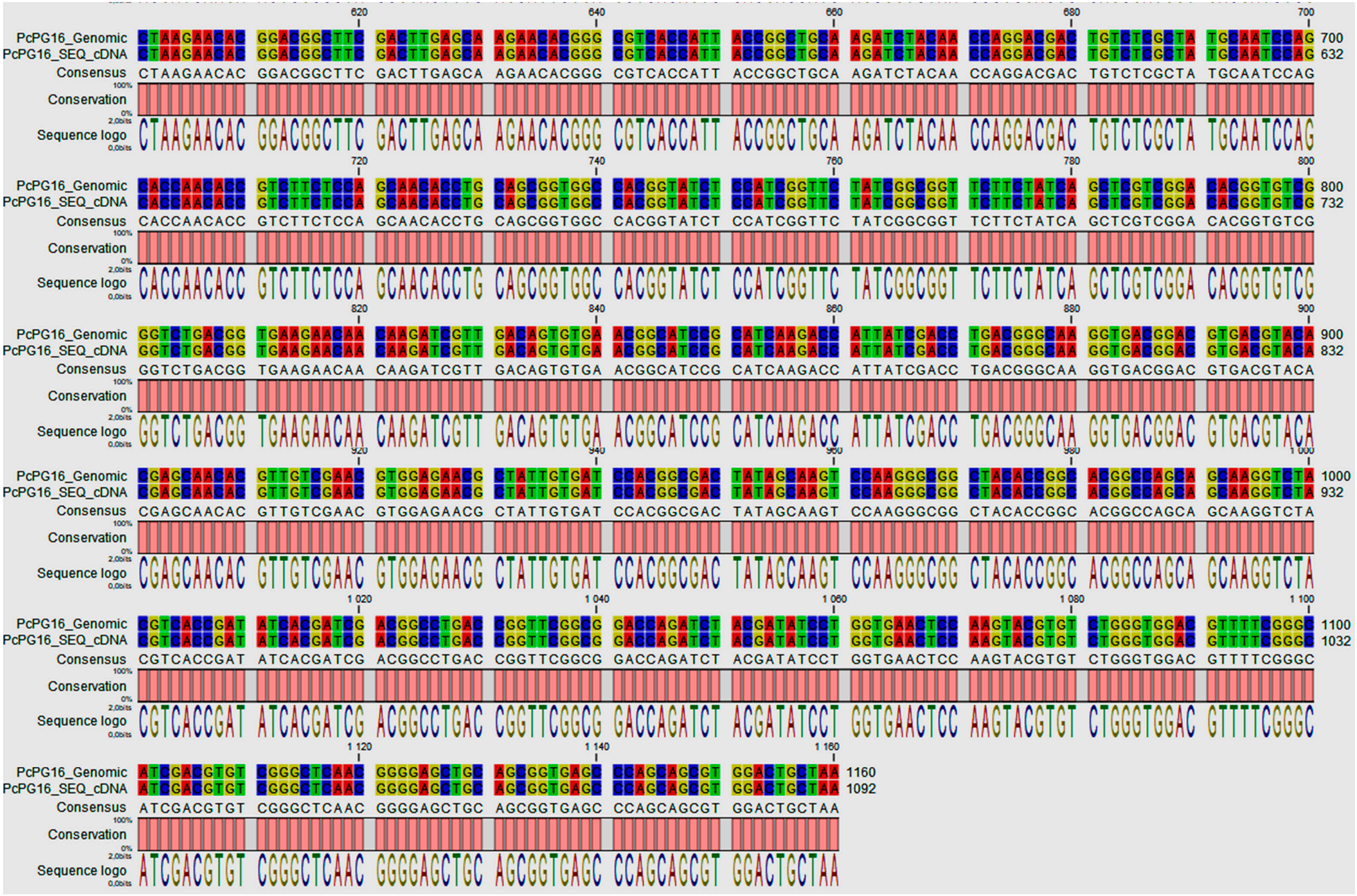
620 640 660 680 700
PcPG16_Genomic CTAAGAACAC GGACGGCTTC GACTTGAGCA AGAACACGGG CGTCACCATT ACCGGCTGCA AGATCTACAA CCAGGACGAC TGTCTCGCTA TGCAATCCAG 700
PcPG16_SEQ_cDNA CTAAGAACAC GGACGGCTTC GACTTGAGCA AGAACACGGG CGTCACCATT ACCGGCTGCA AGATCTACAA CCAGGACGAC TGTCTCGCTA TGCAATCCAG 632
Consensus CTAAGAACAC GGACGGCTTC GACTTGAGCA AGAACACGGG CGTCACCATT ACCGGCTGCA AGATCTACAA CCAGGACGAC TGTCTCGCTA TGCAATCCAG
Conservation
Sequence logo
720 740 760 780 800
PcPG16_Genomic CACCAACACC GTCTTCTCCA GCAACACCTG CAGCGGTGGC CACGGTATCT CCATCGGTTC TATCGGCGGT TCTTCTATCA GCTCGTCGGA CACGGTGTCG 800
PcPG16_SEQ_cDNA CACCAACACC GTCTTCTCCA GCAACACCTG CAGCGGTGGC CACGGTATCT CCATCGGTTC TATCGGCGGT TCTTCTATCA GCTCGTCGGA CACGGTGTCG 732
Consensus CACCAACACC GTCTTCTCCA GCAACACCTG CAGCGGTGGC CACGGTATCT CCATCGGTTC TATCGGCGGT TCTTCTATCA GCTCGTCGGA CACGGTGTCG
Conservation
Sequence logo
820 840 860 880 900
PcPG16_Genomic GGTCTGACGG TGAAGAACAA CAAGATCGTT GACAGTGTGA ACGGCATCCG CATCAAGACC ATTATCGACC TGACGGGCAA GGTGACGGAC GTGACGTACA 900
PcPG16_SEQ_cDNA GGTCTGACGG TGAAGAACAA CAAGATCGTT GACAGTGTGA ACGGCATCCG CATCAAGACC ATTATCGACC TGACGGGCAA GGTGACGGAC GTGACGTACA 832
Consensus GGTCTGACGG TGAAGAACAA CAAGATCGTT GACAGTGTGA ACGGCATCCG CATCAAGACC ATTATCGACC TGACGGGCAA GGTGACGGAC GTGACGTACA
Conservation
Sequence logo
920 940 960 980 1 000
PcPG16_Genomic CGAGCAACAC GTTGTCGAAC GTGGAGAACG CTATTGTGAT CCACGGCGAC TATAGCAAGT CCAAGGGCGG CTACACCGGC ACGGCCAGCA GCAAGGTCTA 1000
PcPG16_SEQ_cDNA CGAGCAACAC GTTGTCGAAC GTGGAGAACG CTATTGTGAT CCACGGCGAC TATAGCAAGT CCAAGGGCGG CTACACCGGC ACGGCCAGCA GCAAGGTCTA 932
Consensus CGAGCAACAC GTTGTCGAAC GTGGAGAACG CTATTGTGAT CCACGGCGAC TATAGCAAGT CCAAGGGCGG CTACACCGGC ACGGCCAGCA GCAAGGTCTA
Conservation
Sequence logo
1 020 1 040 1 060 1 080 1 100
PcPG16_Genomic CGTCACCGAT ATCACGATCG ACGGCCTGAC CGGTTCGGCG GACCAGATCT ACGATATCCT GGTGAACTCC AAGTACGTGT CTGGGTGGAC GTTTTCGGGC 1100
PcPG16_SEQ_cDNA CGTCACCGAT ATCACGATCG ACGGCCTGAC CGGTTCGGCG GACCAGATCT ACGATATCCT GGTGAACTCC AAGTACGTGT CTGGGTGGAC GTTTTCGGGC 1032
Consensus CGTCACCGAT ATCACGATCG ACGGCCTGAC CGGTTCGGCG GACCAGATCT ACGATATCCT GGTGAACTCC AAGTACGTGT CTGGGTGGAC GTTTTCGGGC
Conservation
Sequence logo
1 120 1 140 1 160
PcPG16_Genomic ATCGACGTGT CGGGCTCAAC GGGGAGCTGC AGCGGTGAGC CCAGCAGCGT GGACTGCTAA 1160
PcPG16_SEQ_cDNA ATCGACGTGT CGGGCTCAAC GGGGAGCTGC AGCGGTGAGC CCAGCAGCGT GGACTGCTAA 1092
Consensus ATCGACGTGT CGGGCTCAAC GGGGAGCTGC AGCGGTGAGC CCAGCAGCGT GGACTGCTAA
Conservation
Sequence logo

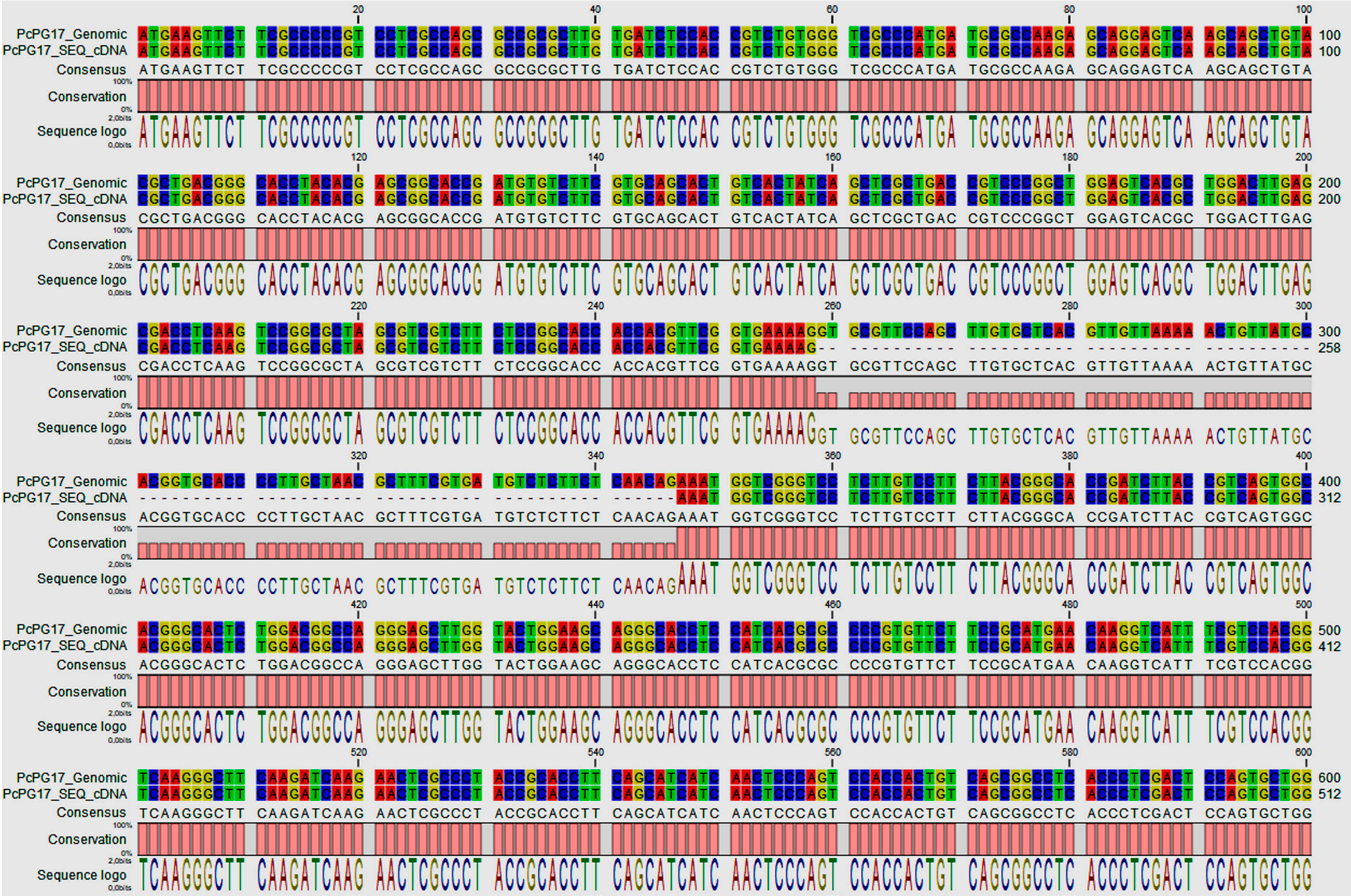

PcPG17_Genomic 100
PcPG17_SEQ_cDNA 100
Consensus ATGAAGTTCT TCGCCCCCGT CCTCGCCAGC GCCGCGCTTG TGATCTCCAC CGTCTGTGGG TCGCCCATGA TGCGCCAAGA GCAGGAGTCA AGCAGCTGTA
Conservation
Sequence logo
PcPG17_Genomic 200
PcPG17_SEQ_cDNA 200
Consensus CGCTGACGGG CACCTACACG AGCGGCACCG ATGTGTCTTC GTGCAGCACT GTCACTATCA GCTCGCTGAC CGTCCCGGCT GGAGTCACGC TGGACTTGAG
PcPG17_Genomic 300
PcPG17_SEQ_cDNA 258
Consensus CGACCTCAAG TCCGGCGCTA GCGTCGTCTT CTCCGGCACC ACCACGTTCG GTGAAAAGGT GCGTTCCAGC TTGTGCTCAC GTTGTTAAAA ACTGTTATGC
PcPG17_Genomic 400
PcPG17_SEQ_cDNA 312
Consensus ACGGTGCACC CCTTGCTAAC GCTTTCGTGA TGTCTCTTCT CAACAGAAAT GGTCGGGTCC TCTTGTCCTT CTTACGGGCA CCGATCTTAC CGTCAGTGGC
PcPG17_Genomic 500
PcPG17_SEQ_cDNA 412
Consensus ACGGGCACTC TGGACGGCCA GGGAGCTTGG TACTGGAAGC AGGGCACCTC CATCACGCGC CCCGTGTTCT TCCGCATGAA CAAGGTCATT TCGTCCACGG
PcPG17_Genomic 600
PcPG17_SEQ_cDNA 512
Consensus TCAAGGGCTT CAAGATCAAG AACTCGCCCT ACCGCACCTT CAGCATCATC AACTCCCAGT CCACCACTGT CAGCGGCCTC ACCCTCGACT CCAGTGCTGG

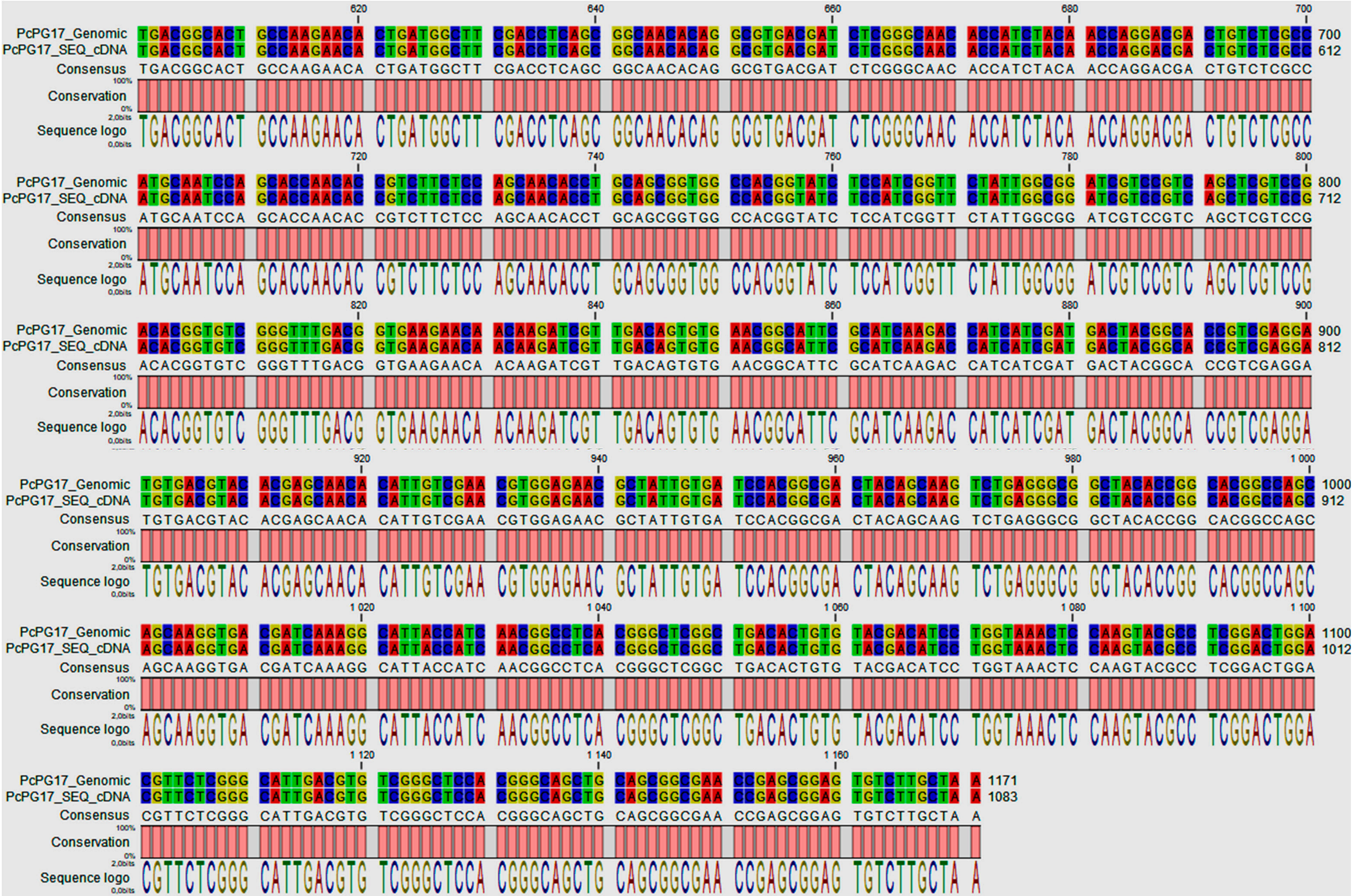
PcPG17_Genomic
PcPG17_SEQ_cDNA
Consensus
Conservation
Sequence logo
TGACGGCACT GCCAAGAACA CTGATGGCTT CGACCTCAGC GGCAACACAG GCGTGACGAT CTCGGGCAAC ACCATCTACA ACCAGGACGA CTGTCTCGCC 700 612
ATGCAATCCA GCACCAACAC CGTCTTCTCC AGCAACACCT GCAGCGGTGG CCACGGTATC TCCATCGGTT CTATTGGCGG ATCGTCCGTC AGCTCGTCCG 800 712
ACACGGTGTC GGGTTTGACG GTGAAGAACA ACAAGATCGT TGACAGTGTG AACGGCATTC GCATCAAGAC CATCATCGAT GACTACGGCA CCGTCGAGGA 900 812
TGTGACGTAC ACGAGCAACA CATTGTCGAA CGTGGAGAAC GCTATTGTGA TCCACGGCGA CTACAGCAAG TCTGAGGGCG GCTACACCGG CACGGCCAGC 1000 912
AGCAAGGTGA CGATCAAAGG CATTACCATC AACGGCCTCA CGGGCTCGGC TGACACTGTG TACGACATCC TGGTAAACTC CAAGTACGCC TCGGACTGGA 1100 1012
CGTTCTCGGG CATTGACGTG TCGGGCTCCA CGGGCAGCTG CAGCGGCGAA CCGAGCGGAG TGTCTTGCTA A 1171 1083

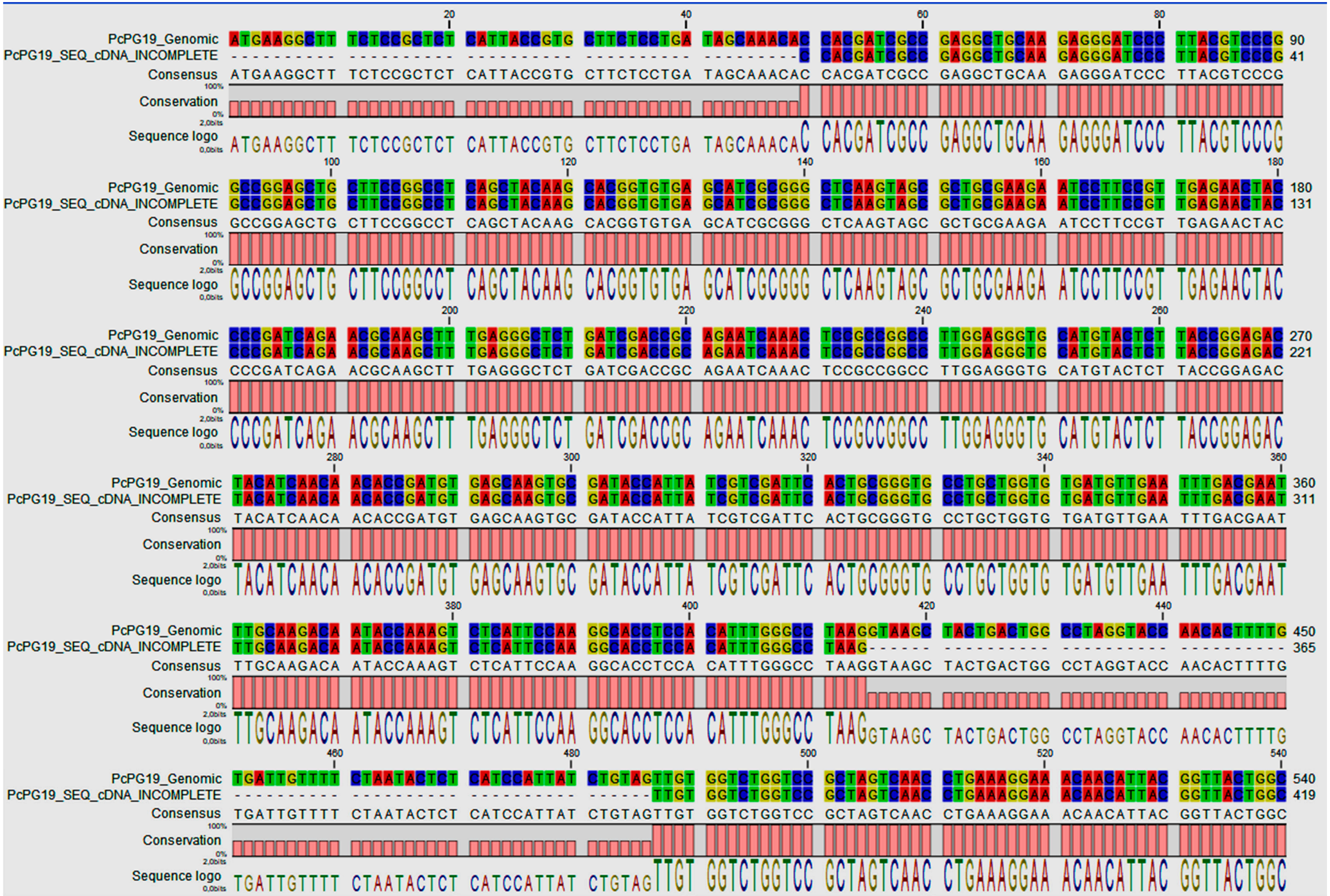
PcPG19_Genomic ATGAAGGCTT TCTCCGCTCT CATTACCGTG CTTCTCCTGA TAGCAAACAC CACGATCGCC GAGGCTGCAA GAGGGATCCC TTACGTCCCG 90
PcPG19_SEQ_cDNA_INCOMPLETE ---------- ---------- ---------- ---------- ---------C CACGATCGCC GAGGCTGCAA GAGGGATCCC TTACGTCCCG 41
Consensus ATGAAGGCTT TCTCCGCTCT CATTACCGTG CTTCTCCTGA TAGCAAACAC CACGATCGCC GAGGCTGCAA GAGGGATCCC TTACGTCCCG
Conservation
Sequence logo
PcPG19_Genomic GCCGGAGCTG CTTCCGGCCT CAGCTACAAG CACGGTGTGA GCATCGCGGG CTCAAGTAGC GCTGCGAAGA ATCCTTCCGT TGAGAACTAC 180
PcPG19_SEQ_cDNA_INCOMPLETE GCCGGAGCTG CTTCCGGCCT CAGCTACAAG CACGGTGTGA GCATCGCGGG CTCAAGTAGC GCTGCGAAGA ATCCTTCCGT TGAGAACTAC 131
Consensus GCCGGAGCTG CTTCCGGCCT CAGCTACAAG CACGGTGTGA GCATCGCGGG CTCAAGTAGC GCTGCGAAGA ATCCTTCCGT TGAGAACTAC
Conservation
Sequence logo
PcPG19_Genomic CCCGATCAGA ACGCAAGCTT TGAGGGCTCT GATCGACCGC AGAATCAAAC TCCGCCGGCC TTGGAGGGTG CATGTACTCT TACCGGAGAC 270
PcPG19_SEQ_cDNA_INCOMPLETE CCCGATCAGA ACGCAAGCTT TGAGGGCTCT GATCGACCGC AGAATCAAAC TCCGCCGGCC TTGGAGGGTG CATGTACTCT TACCGGAGAC 221
Consensus CCCGATCAGA ACGCAAGCTT TGAGGGCTCT GATCGACCGC AGAATCAAAC TCCGCCGGCC TTGGAGGGTG CATGTACTCT TACCGGAGAC
Conservation
Sequence logo
PcPG19_Genomic TACATCAACA ACACCGATGT GAGCAAGTGC GATACCATTA TCGTCGATTC ACTGCGGGTG CCTGCTGGTG TGATGTTGAA TTTGACGAAT 360
PcPG19_SEQ_cDNA_INCOMPLETE TACATCAACA ACACCGATGT GAGCAAGTGC GATACCATTA TCGTCGATTC ACTGCGGGTG CCTGCTGGTG TGATGTTGAA TTTGACGAAT 311
Consensus TACATCAACA ACACCGATGT GAGCAAGTGC GATACCATTA TCGTCGATTC ACTGCGGGTG CCTGCTGGTG TGATGTTGAA TTTGACGAAT
Conservation
Sequence logo
PcPG19_Genomic TTGCAAGACA ATACCAAAGT CTCATTCCAA GGCACCTCCA CATTTGGGCC TAAGGTAAGC TACTGACTGG CCTAGGTACC AACACTTTTG 450
PcPG19_SEQ_cDNA_INCOMPLETE TTGCAAGACA ATACCAAAGT CTCATTCCAA GGCACCTCCA CATTTGGGCC TAAG------ ---------- ---------- ---------- 365
Consensus TTGCAAGACA ATACCAAAGT CTCATTCCAA GGCACCTCCA CATTTGGGCC TAAGGTAAGC TACTGACTGG CCTAGGTACC AACACTTTTG
Conservation
Sequence logo
PcPG19_Genomic TGATTGTTTT CTAATACTCT CATCCATTAT CTGTAGTTGT GGTCTGGTCC GCTAGTCAAC CTGAAAGGAA ACAACATTAC GGTTACTGGC 540
PcPG19_SEQ_cDNA_INCOMPLETE ---------- ---------- ---------- ------TTGT GGTCTGGTCC GCTAGTCAAC CTGAAAGGAA ACAACATTAC GGTTACTGGC 419
Consensus TGATTGTTTT CTAATACTCT CATCCATTAT CTGTAGTTGT GGTCTGGTCC GCTAGTCAAC CTGAAAGGAA ACAACATTAC GGTTACTGGC
Conservation
Sequence logo

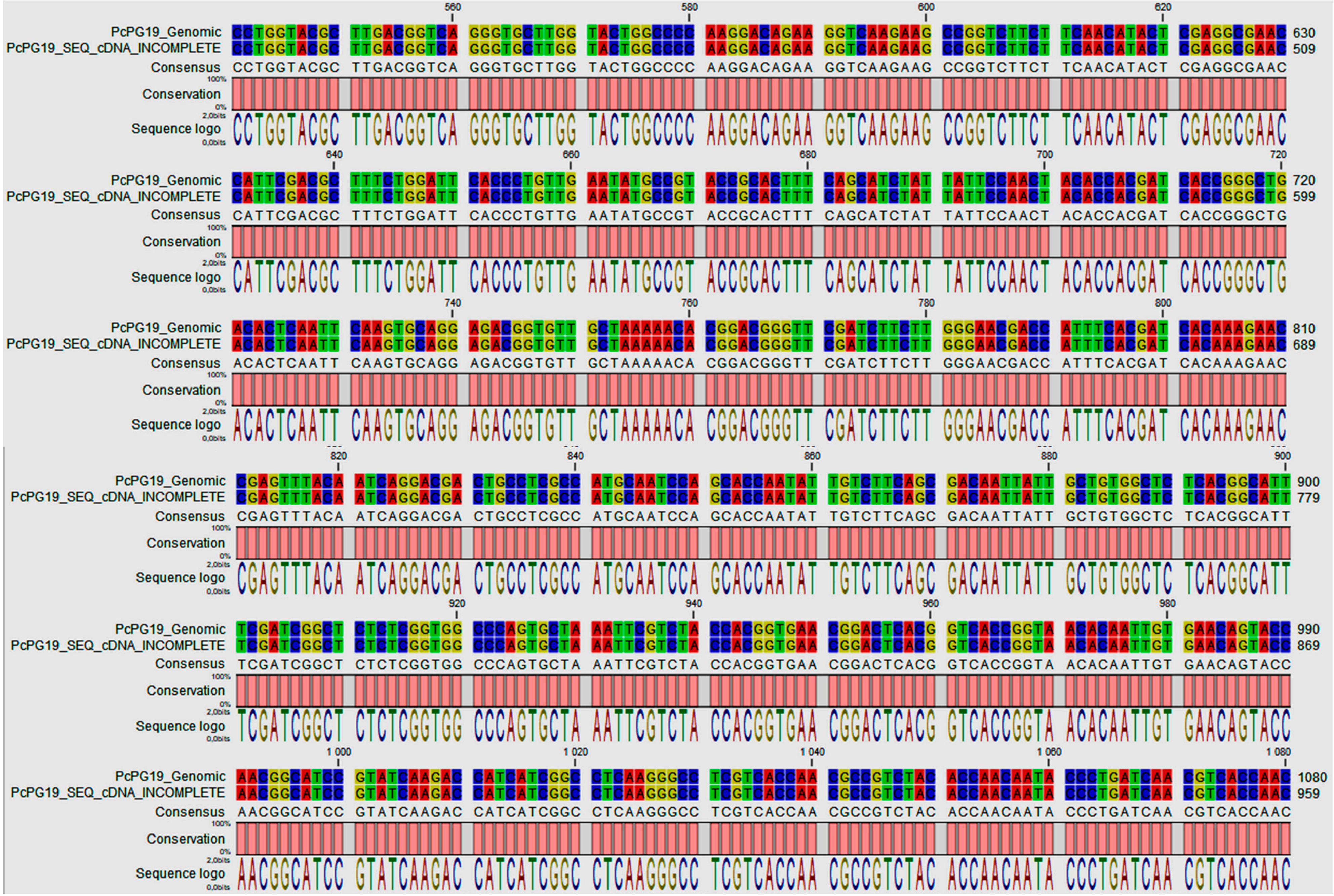
560 580 600 620
PcPG19_Genomic CCTGGTACGC TTGACGGTCA GGGTGCTTGG TACTGGCCCC AAGGACAGAA GGTCAAGAAG CCGGTCTTCT TCAACATACT CGAGGCGAAC 630
PcPG19_SEQ_cDNA_INCOMPLETE CCTGGTACGC TTGACGGTCA GGGTGCTTGG TACTGGCCCC AAGGACAGAA GGTCAAGAAG CCGGTCTTCT TCAACATACT CGAGGCGAAC 509
Consensus CCTGGTACGC TTGACGGTCA GGGTGCTTGG TACTGGCCCC AAGGACAGAA GGTCAAGAAG CCGGTCTTCT TCAACATACT CGAGGCGAAC
Conservation
Sequence logo
640 660 680 700 720
PcPG19_Genomic CATTCGACGC TTTCTGGATT CACCCTGTTG AATATGCCGT ACCGCACTTT CAGCATCTAT TATTCCAACT ACACCACGAT CACCGGGCTG 720
PcPG19_SEQ_cDNA_INCOMPLETE CATTCGACGC TTTCTGGATT CACCCTGTTG AATATGCCGT ACCGCACTTT CAGCATCTAT TATTCCAACT ACACCACGAT CACCGGGCTG 599
Consensus CATTCGACGC TTTCTGGATT CACCCTGTTG AATATGCCGT ACCGCACTTT CAGCATCTAT TATTCCAACT ACACCACGAT CACCGGGCTG
Conservation
Sequence logo
740 760 780 800
PcPG19_Genomic ACACTCAATT CAAGTGCAGG AGACGGTGTT GCTAAAAACA CGGACGGGTT CGATCTTCTT GGGAACGACC ATTTCACGAT CACAAAGAAC 810
PcPG19_SEQ_cDNA_INCOMPLETE ACACTCAATT CAAGTGCAGG AGACGGTGTT GCTAAAAACA CGGACGGGTT CGATCTTCTT GGGAACGACC ATTTCACGAT CACAAAGAAC 689
Consensus ACACTCAATT CAAGTGCAGG AGACGGTGTT GCTAAAAACA CGGACGGGTT CGATCTTCTT GGGAACGACC ATTTCACGAT CACAAAGAAC
Conservation
Sequence logo
820 840 860 880 900
PcPG19_Genomic CGAGTTTACA ATCAGGACGA CTGCCTCGCC ATGCAATCCA GCACCAATAT TGTCTTCAGC GACAATTATT GCTGTGGCTC TCACGGCATT 900
PcPG19_SEQ_cDNA_INCOMPLETE CGAGTTTACA ATCAGGACGA CTGCCTCGCC ATGCAATCCA GCACCAATAT TGTCTTCAGC GACAATTATT GCTGTGGCTC TCACGGCATT 779
Consensus CGAGTTTACA ATCAGGACGA CTGCCTCGCC ATGCAATCCA GCACCAATAT TGTCTTCAGC GACAATTATT GCTGTGGCTC TCACGGCATT
Conservation
Sequence logo
920 940 960 980
PcPG19_Genomic TCGATCGGCT CTCTCGGTGG CCCAGTGCTA AATTCGTCTA CCACGGTGAA CGGACTCACG GTCACCGGTA ACACAATTGT GAACAGTACC 990
PcPG19_SEQ_cDNA_INCOMPLETE TCGATCGGCT CTCTCGGTGG CCCAGTGCTA AATTCGTCTA CCACGGTGAA CGGACTCACG GTCACCGGTA ACACAATTGT GAACAGTACC 869
Consensus TCGATCGGCT CTCTCGGTGG CCCAGTGCTA AATTCGTCTA CCACGGTGAA CGGACTCACG GTCACCGGTA ACACAATTGT GAACAGTACC
Conservation
Sequence logo
1 000 1 020 1 040 1 060 1 080
PcPG19_Genomic AACGGCATCC GTATCAAGAC CATCATCGGC CTCAAGGGCC TCGTCACCAA CGCCGTCTAC ACCAACAATA CCCTGATCAA CGTCACCAAC 1080
PcPG19_SEQ_cDNA_INCOMPLETE AACGGCATCC GTATCAAGAC CATCATCGGC CTCAAGGGCC TCGTCACCAA CGCCGTCTAC ACCAACAATA CCCTGATCAA CGTCACCAAC 959
Consensus AACGGCATCC GTATCAAGAC CATCATCGGC CTCAAGGGCC TCGTCACCAA CGCCGTCTAC ACCAACAATA CCCTGATCAA CGTCACCAAC
Conservation
Sequence logo

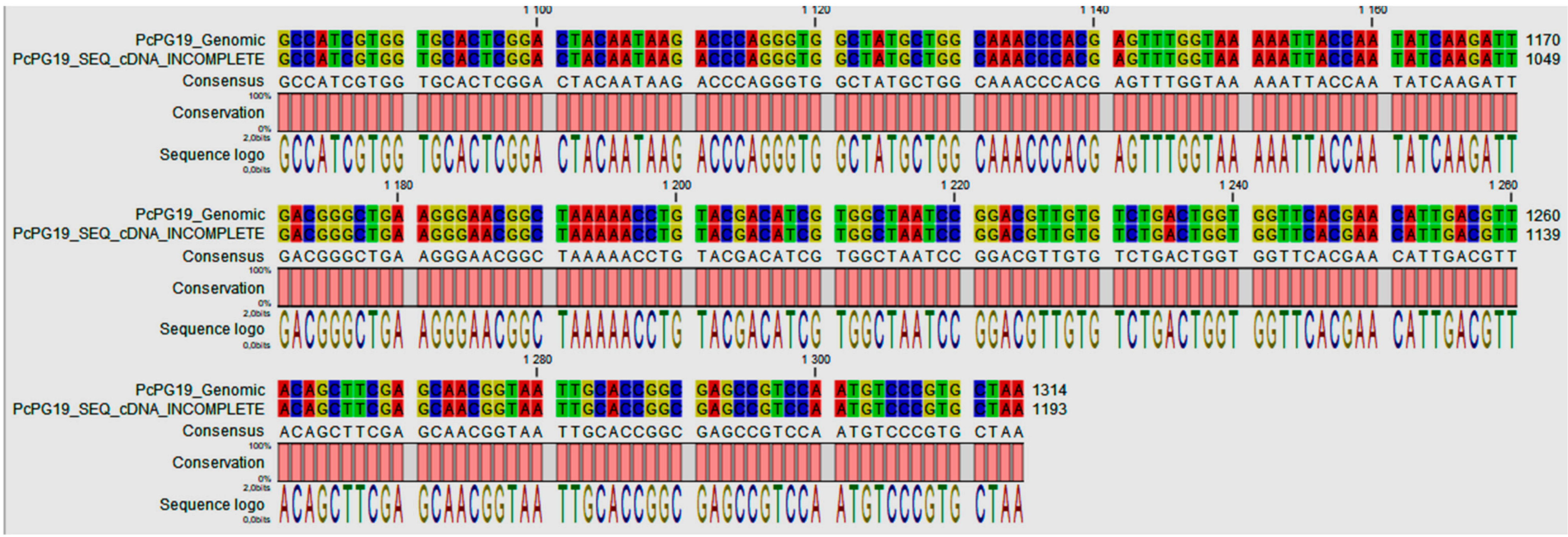
PcPG19_Genomic
PcPG19_SEQ_cDNA_INCOMPLETE
Consensus
Conservation
Sequence logo
1 100
1 120
1 140
1 160
GCCATCGTGG TGCACTCGGA CTACAATAAG ACCCAGGGTG GCTATGCTGG CAAACCCACG AGTTTGGTAA AAATTACCAA TATCAAGATT
1170
1049
1 180
1 200
1 220
1 240
1 260
GACGGGCTGA AGGGAACGGC TAAAAACCTG TACGACATCG TGGCTAATCC GGACGTTGTG TCTGACTGGT GGTTCACGAA CATTGACGTT
1260
1139
1 280
1 300
ACAGCTTCGA GCAACGGTAA TTGCACCGGC GAGCCGTCCA ATGTCCCGTG CTAA
1314
1193

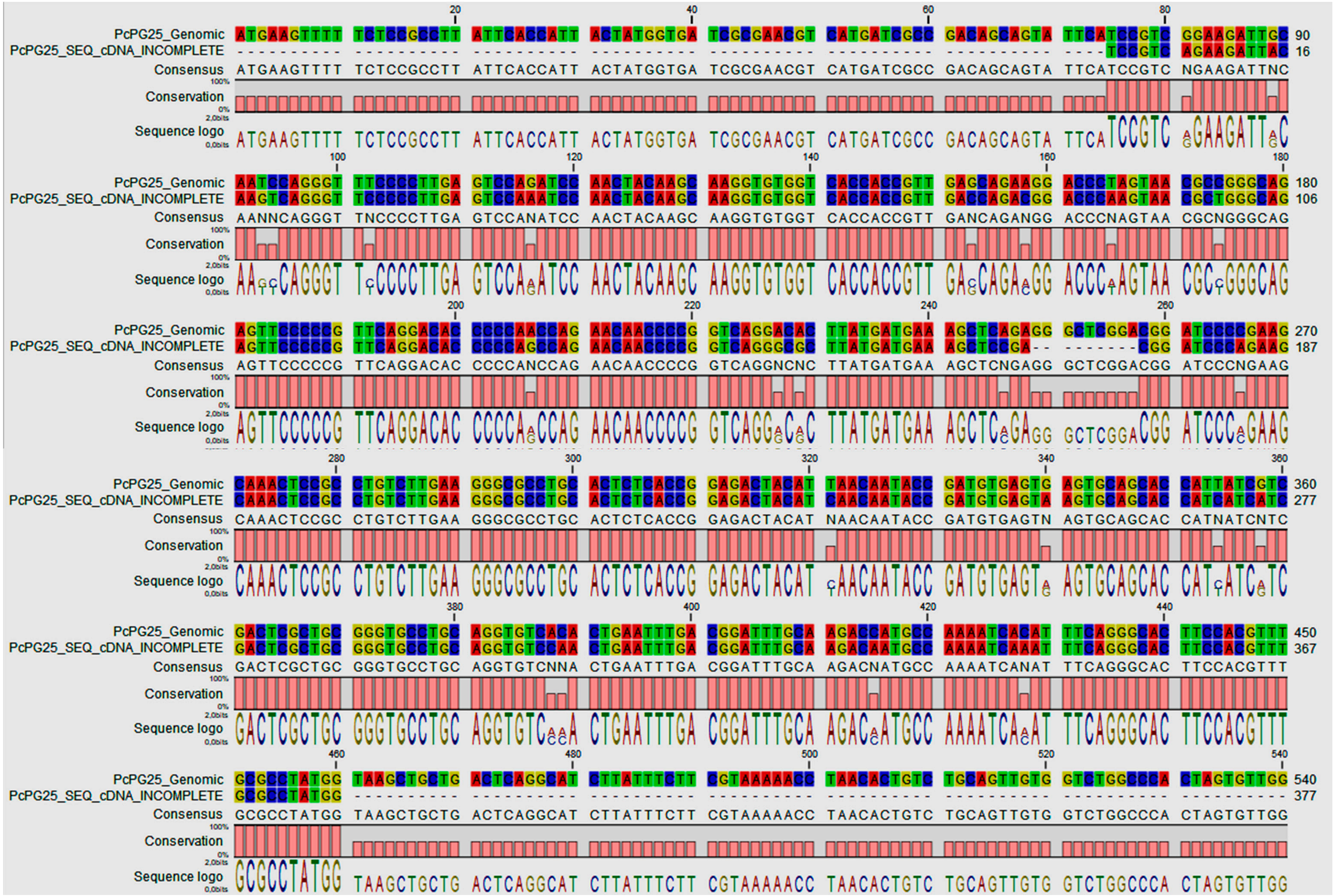
PcPG25_Genomic
PcPG25_SEQ_cDNA_INCOMPLETE
Consensus
Conservation
Sequence logo
ATGAAGTTTT TCTCCGCCTT ATTCACCATT ACTATGGTGA TCGCGAACGT CATGATCGCC GACAGCAGTA TTCATCCGTC NGAAGATTNC 90 16
AANNCAGGGT TNCCCCTTGA GTCCANATCC AACTACAAGC AAGGTGTGGT CACCACCGTT GANCAGANGG ACCCNAGTAA CGCNGGGCAG 180 106
AGTTCCCCCG TTCAGGACAC CCCCANCCAG AACAACCCCG GTCAGGNCNC TTATGATGAA AGCTCNGAGG GCTCGGACGG ATCCCNGAAG 270 187
CAAACTCCGC CTGTCTTGAA GGGCGCCTGC ACTCTCACCG GAGACTACAT NAACAATACC GATGTGAGTN AGTGCAGCAC CATNATCNTC 360 277
GACTCGCTGC GGGTGCCTGC AGGTGTCNNA CTGAATTTGA CGGATTTGCA AGACNATGCC AAAATCANAT TTCAGGGCAC TTCCACGTTT 450 367
GCGCCTATGG TAAGCTGCTG ACTCAGGCAT CTTATTTCTT CGTAAAAACC TAACACTGTC TGCAGTTGTG GTCTGGCCCA CTAGTGTTGG 540 377

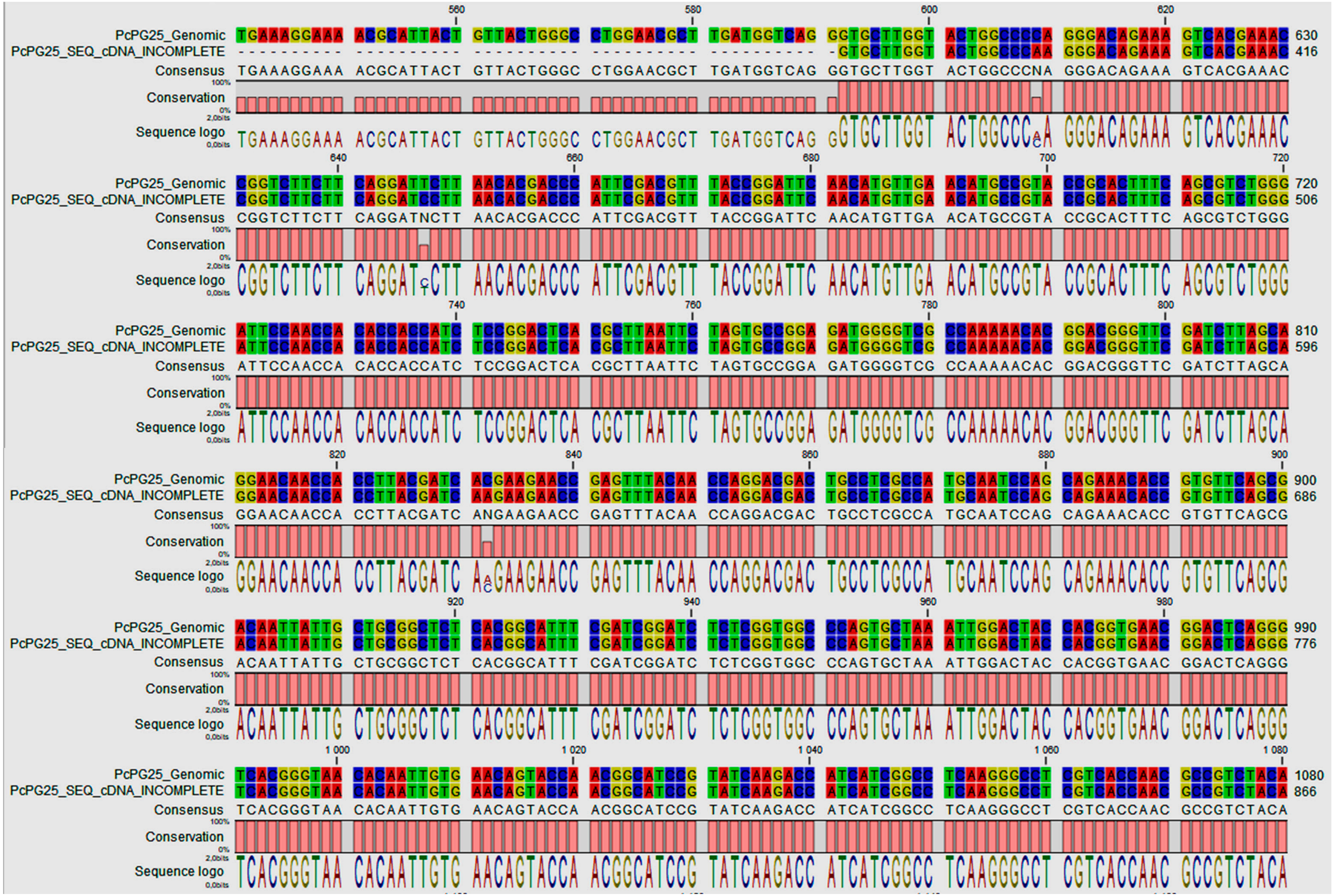

560 580 600 620
PcPG25_Genomic TGAAAGGAAA ACGCATTACT GTTACTGGGC CTGGAACGCT TGATGGTCAG GGTGCTTGGT ACTGGCCCCA GGGACAGAAA GTCACGAAAC 630
PcPG25_SEQ_cDNA_INCOMPLETE ---------- ---------- ---------- ---------- ---------- -GTGCTTGGT ACTGGCCCAA GGGACAGAAA GTCACGAAAC 416
Consensus TGAAAGGAAA ACGCATTACT GTTACTGGGC CTGGAACGCT TGATGGTCAG GGTGCTTGGT ACTGGCCCNA GGGACAGAAA GTCACGAAAC
Conservation
Sequence logo
640 660 680 700 720
PcPG25_Genomic CGGTCTTCTT CAGGATTCTT AACACGACCC ATTCGACGTT TACCGGATTC AACATGTTGA ACATGCCGTA CCGCACTTTC AGCGTCTGGG 720
PcPG25_SEQ_cDNA_INCOMPLETE CGGTCTTCTT CAGGATCCTT AACACGACCC ATTCGACGTT TACCGGATTC AACATGTTGA ACATGCCGTA CCGCACTTTC AGCGTCTGGG 506
Consensus CGGTCTTCTT CAGGATNCTT AACACGACCC ATTCGACGTT TACCGGATTC AACATGTTGA ACATGCCGTA CCGCACTTTC AGCGTCTGGG
Conservation
Sequence logo
740 760 780 800
PcPG25_Genomic ATTCCAACCA CACCACCATC TCCGGACTCA CGCTTAATTC TAGTGCCGGA GATGGGGTCG CCAAAAACAC GGACGGGTTC GATCTTAGCA 810
PcPG25_SEQ_cDNA_INCOMPLETE ATTCCAACCA CACCACCATC TCCGGACTCA CGCTTAATTC TAGTGCCGGA GATGGGGTCG CCAAAAACAC GGACGGGTTC GATCTTAGCA 596
Consensus ATTCCAACCA CACCACCATC TCCGGACTCA CGCTTAATTC TAGTGCCGGA GATGGGGTCG CCAAAAACAC GGACGGGTTC GATCTTAGCA
Conservation
Sequence logo
820 840 860 880 900
PcPG25_Genomic GGAACAACCA CCTTACGATC ACGAAGAACC GAGTTTACAA CCAGGACGAC TGCCTCGCCA TGCAATCCAG CAGAAACACC GTGTTCAGCG 900
PcPG25_SEQ_cDNA_INCOMPLETE GGAACAACCA CCTTACGATC AAGAAGAACC GAGTTTACAA CCAGGACGAC TGCCTCGCCA TGCAATCCAG CAGAAACACC GTGTTCAGCG 686
Consensus GGAACAACCA CCTTACGATC ANGAAGAACC GAGTTTACAA CCAGGACGAC TGCCTCGCCA TGCAATCCAG CAGAAACACC GTGTTCAGCG
Conservation
Sequence logo
920 940 960 980
PcPG25_Genomic ACAATTATTG CTGCGGCTCT CACGGCATTT CGATCGGATC TCTCGGTGGC CCAGTGCTAA ATTGGACTAC CACGGTGAAC GGACTCAGGG 990
PcPG25_SEQ_cDNA_INCOMPLETE ACAATTATTG CTGCGGCTCT CACGGCATTT CGATCGGATC TCTCGGTGGC CCAGTGCTAA ATTGGACTAC CACGGTGAAC GGACTCAGGG 776
Consensus ACAATTATTG CTGCGGCTCT CACGGCATTT CGATCGGATC TCTCGGTGGC CCAGTGCTAA ATTGGACTAC CACGGTGAAC GGACTCAGGG
Conservation
Sequence logo
1 000 1 020 1 040 1 060 1 080
PcPG25_Genomic TCACGGGTAA CACAATTGTG AACAGTACCA ACGGCATCCG TATCAAGACC ATCATCGGCC TCAAGGGCCT CGTCACCAAC GCCGTCTACA 1080
PcPG25_SEQ_cDNA_INCOMPLETE TCACGGGTAA CACAATTGTG AACAGTACCA ACGGCATCCG TATCAAGACC ATCATCGGCC TCAAGGGCCT CGTCACCAAC GCCGTCTACA 866
Consensus TCACGGGTAA CACAATTGTG AACAGTACCA ACGGCATCCG TATCAAGACC ATCATCGGCC TCAAGGGCCT CGTCACCAAC GCCGTCTACA
Conservation
Sequence logo

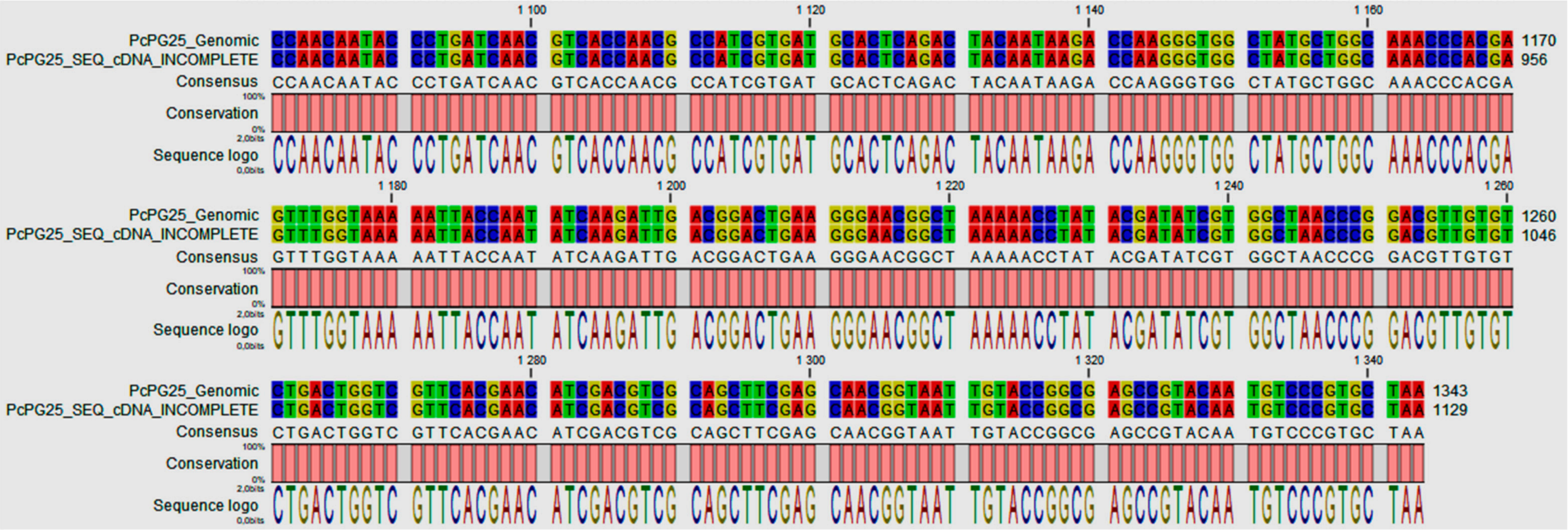

Figure S3. Pairwise alignment (zoomed in) of intron-containing PG genomic sequences with their corresponding coding sequences. The genomic sequences of the introns are at the top and the coding sequences are below the genomic sequences. The consensus sequence is shown below the alignment and the sequence conservation is indicated by the pink bars.

Table S4. Representation of the intron exon boundaries of polygalacturonase genes from *Phytophthora cinnamomi*-GKB4 genome (accession: JAFJYM000000000) using Augustus for gene prediction.

Gene name	Gene	Start codon	CDS1	CDS2	CDS3	Stop codon	No. of exons
pcpg1	3159477-3160685	3160683-3160685	3159477-3160496	3160563-3160685	none	3064418-3064420	2
Pcpg2	3159477-3160685	3160683-3160685	3159477-3160685	none	none	3159477-3159479	1
Pcpg3	687409-688584	688582-688584	687409-688584	none	none	687409-687411	1
Pcpg4	699645-700820	699645-699647	699645-700820	none	none	700818-700820	1
Pcpg5	3117095-3118339	3118337-3118339	3117095-3118339	none	none	3117095-3117097	1
Pcpg6	3062862-3063947	3062862-3062864	3062862-3063947	none	none	3063945-3063947	1
pcpg7	690379-691554	691552-691554	690379-691554	none	none	690379-690381	1
pcpg8	3060266-3061351	3061349-3061351	3060266-3061351	none	none	3060266-3060268	1
Pcpg9	696762-697853	696762-696764	696762-697853	none	none	697851-697853	1
Pcpg10	3175783-3177147	3177145-3177147	3175783-3177147	none	none	3175783-3175785	1
Pcpg11	3179776-3181194	3181192-3181194	3179776-3181194	none	none	3179776-3179778	1
Pcpg12	3183124-3184542	3183124-3183126	3183124-3184542	none	none	3184540-3184542	1
Pcpg13	108265-109683	109681-109683	108265-109683	none	none	108265-108267	1
Pcpg14	111608-113050	111608-111610	111608-113050	none	none	113048-113050	1

**Table S4. Representation of the intron exon boundaries of polygalacturonase genes from *Phytophthora cinnamomi*-GKB4 genome (accession: JAFJYM000000000) using Augustus for gene prediction.**							
**Gene name**	**Gene**	**Start codon**	**CDS1**	**CDS2**	**CDS3**	**Stop codon**	**No. of exons**
Pcpg16	3054273-3055432	3054273-3054275	3054273-3054539	3054608-3055432	none	3055430-3055432	2
Pcpg17	3056638-3057808	3056638-3056640	3056638-3056895	3056984-3057808	none	3057806-3057808	2
Pcpg19	1649208-1650521	1650519-1650521	1649208-1650035	1650108-1650521	none	1649208-1649210	2
Pcpg22	444730-446301	446299-446301	444730-446301	none	none	444730-444732	1
Pcpg25	422021-423363	423361-423363	422021-422773	422905-423363	none	422021-422023	2

Table S5. Protein sequence similarity of full-length and partial putative polygalacturonase proteins of *Phytophthora cinnamomi* to PG proteins from *Phytophthora spp.*, based on BLASTP analyses.

New gene name	Query ID from the South African genome	NCBI accession	Query coverage	E-value	% Identity	Species description	Reference
PcPG1	OL334941	AAN05466	99%	0.0	90.82%	Polygalacturonase 7 [*P. cinnamomi*]	[1]
PcPG2	OL334942	AAN05466	99%	0.0	96.86%	Polygalacturonase 7 [*P. cinnamomi*]	[1]
PcPG3	OL334943	KUF94019	99%	0.0	84.65%	Endopolygalacturonase 2 [*P. nicotianae*]	[2]
PcPG4	OL334944	KUF94019	99%	0.0	84.65%	Endopolygalacturonase 2 [*P. nicotianae*]	[2]
PcPG5	OL334945	AAN05466	99%	0.0	99.52%	Polygalacturonase 7 [*P. cinnamomi*]	[1]
PcPG6	OL334946	AAN34613	99%	0.0	100%	Polygalacturonase 2 [*P. cinnamomi*]	[1]
PcPG7	OL334947	KUF94019	99%	0.0	79.69%	Endopolygalacturonase 2 [*P. nicotianae*]	[2]
PcPG8	OL334948	AAN34614	99%	0.0	99.72%	Polygalacturonase 3 [*P. cinnamomi*]	[1]
PcPG9	OL334949	POM75086	99%	0.0	78.51%	Polygalacturonase [*Phytophthora palmivora var. palmivora*]	[3]
PcPG10	OL334950	AAN05465	99%	0.0	99.78%	Polygalacturonase 10 [*P. cinnamomi*]	[1]
PcPG11	OL334951	KUF84330	96%	0.0	73.03%	Polygalacturonase [*P. nicotianae*]	Unpublished
PcPG12	OL334952	KUF84330	96%	0.0	73.03%	Polygalacturonase [*P. nicotianae*]	Unpublished

Table S5. Protein sequence similarity of full-length and partial putative polygalacturonase proteins of *Phytophthora cinnamomi*, to PGs from *Phytophthora spp.* based on BLASTP analyses.

New gene name	Query ID from JGI	NCBI accession	Query coverage	E-value	% Identity	Species description	Reference
PcPG13	OL334953	KUF84330	96%	0.0	73.03%	Polygalacturonase [*P. nicotianae*]	Unpublished
PcPG14	OL334954	KUF84330	96%	0.0	71.77%	Polygalacturonase [*P. nicotianae*]	Unpublished
PcPG15	OL334955	AAN05468	99%	0.0	100.00%	Polygalacturonase 9 [*P. cinnamomi*]	[1]
PcPG16	OL334956	AAN34616	99%	0.0	99.72%	Polygalacturonase 6 [*P. cinnamomi*]	[1]
PcPG17	OL334957	AAN34616	99%	0.0	87.88%	Polygalacturonase 6 [*P. cinnamomi*]	[1]
PcPG18	OL334958	AAN34612	99%	0.0	100%	polygalacturonase 1 [*P. cinnamomi*]	[1]
PcPG19	OL334959	AAN05463	99%	0.0	100%	Polygalacturonase 16 [*P. cinnamomi*]	[1]
PcPG20	OL334960	AAN34615	99%	0.0	100%	Polygalacturonase 4 [*P. cinnamomi*]	[1]
PcPG21	OL334961	AAN05458	99%	0.0	100%	Polygalacturonase 19 [*P. cinnamomi*]	[1]
PcPG22	OL334962	ADN18856	62%	0.0	86.89%	Polygalacturonase 12 [*Phytophthora capsici*]	Unpublished
PcPG23	OL334963	AAN05465.	99%	0.0	99.75%	Polygalacturonase [*P. cinnamomi*]	[1]
PcPG24	OL334964	AAN05458	66%	0.0	95.19%	Polygalacturonase 19 [*P. cinnamomi*]	[1]
PcPG25	OL334965	AAN05464	87%	0.0	80.68%	Polygalacturonase 17 [*P. cinnamomi*]	[1]
PcPG26	OL334966	AAN05466	99%	0.0	99.44%	Polygalacturonase 7 [*P. cinnamomi*]	[1]

Table S5. Protein sequence similarity of full-length and partial putative polygalacturonase proteins of *Phytophthora cinnamomi*, to PGs from *Phytophthora spp.* based on BLASTP analyses.

New gene name	Query ID from JGI	NCBI accession	Query coverage	E-value	% Identity	Species description	Reference
PcPG27	OL334967	AAN05466	99%	2e-124	66.26%	Polygalacturonase 7 [*P. cinnamomi*]	[1]
PcPG28	OL334968	POM75086	96%	7e-89	75.84%	Polygalacturonase [*P. palmivora var. palmivora*]	[3]
PcPG29	OL334969	XP_009520006	98%	3e-83	83.87%	Polygalacturonase [*Phytophthora sojae*]	[4]
PcPG30	OL334970	AAN05467	99%	5e-103	100%	Polygalacturonase 8 [*P. cinnamomi*]	[1]
PcPG31	OL334971	AAN05466	99%	2e-113	83.33%	Polygalacturonase 7 [*P. cinnamomi*]	[1]
PcPG32	OL334972	AAN05466	92%	2e-113	78.18%	Polygalacturonase 7 [*P. cinnamomi*]	[1]
PcPG33	OL334973	AAN05466	85%	8e-72	68.51%	Polygalacturonase 7 [*P. cinnamomi*]	[1]
PcPG34	OL334974	AAN05466	99%	3e-62	69.09%	Polygalacturonase 7 [*P. cinnamomi*]	[1]
PcPG35	OL334975	AAN05466	78%	7e-40	63.43%	Polygalacturonase 7 [*P. cinnamomi*]	[1]
PcPG36	OL334976	AAN05466	94%	5e-24	73.97%	Polygalacturonase 7 [*P. cinnamomi*]	[1]
PcPG37	OL334977	AAN05467	65%	1e-19	72.31%	Polygalacturonase 8 [*P. cinnamomi*]	[1]

Table S6. Full-length and partial polygalacturonase sequences from *Phytophthora cinnamomi*.

PG gene ID	Predicted protein (aa)	Signal peptide & position		GH28 domain	Catalytic (G/QDD & G/SHG) & Binding domains (NTD & RIK)	Full-length/ partial
PcPG1	381 aa	Yes	20 – 21	Yes - incomplete	QDD & GHG and NTD & RIK	Full-length
PcPG2	403 aa	Yes	20 – 21	Yes - incomplete	QDD & GHG and NTD & RIK	Full-length
PcPG3	392 aa	Yes	20 – 21	Yes - incomplete	QDD & GHG and NTD & RIK	Full-length
PcPG4	392 aa	Yes	20 – 21	Yes - incomplete	QDD & GHG and NTD & RIK	Full-length
PcPG5	415 aa	Yes	20 – 21	Yes - incomplete	QDD & GHG and NTD & RIK	Full-length
PcPG6	362 aa	Yes	20 – 21	Yes – complete	QDD & GHG and NTD & RIK	Full-length
PcPG7	392 aa	Yes	20 – 21	Yes - incomplete	QDD & GHG and NTD & RIK	Full-length
PcPG8	362 aa	Yes	20 – 21	Yes - complete	QDD & GHG and NTD & RIK	Full-length
PcPG9	364 aa	Yes	20 – 21	Yes - incomplete	QDD & GHG and NTD & RIK	Full-length
PcPG10	455 aa	Yes	20 – 21	Yes - incomplete	QDD & GHG and NTD & RIK	Full-length
PcPG11	473 aa	Yes	20 – 21	Yes - complete	QDD & GHG and NTD & RIK	Full-length
PcPG12	473 aa	Yes	20 – 21	Yes - complete	QDD & GHG and NTD & RIK	Full-length
PcPG13	473 aa	Yes	20 – 21	Yes - complete	QDD & GHG and NTD & RIK	Full-length
PcPG14	481 aa	Yes	20 – 21	Yes - complete	QDD & GHG and NTD & RIK	Full-length
PcPG15	356 aa	No[a]		____		Partial
PcPG16	364 aa	Yes	20 – 21	Yes – incomplete	QDD & GHG and NTD & RIK	Full-length

The ___ is an indication that the sequence was removed for further analysis, and the blank spaces indicate that further analysis was not conducted for the sequence, and therefore there are no results to record.

Table S6. Full-length and partial polygalacturonase sequences from *Phytophthora cinnamomi*

PG gene ID	Predicted protein (aa)	Signal peptide & position		GH28 domain	Catalytic (G/QDD & G/SHG) & Binding domains (NTD & RIK)	Full-length/ partial
PcPG17	361 aa	Yes	20 – 21	Yes – complete	QDD & GHG and NTD & RIK	Full-length
PcPG18	349 aa	No[a]		____		Partial
PcPG19	414 aa	Yes	22 – 23	Yes – incomplete	QDD & SHG and NTD & RIK	Full-length
PcPG20	340 aa	No[a]		____		Partial
PcPG21	328 aa	No[c]		____		Partial
PcPG22	524 aa	Yes	19 – 20	Yes – incomplete	QDD & GHG and NTD & RIK	Full-length
PcPG23	400 aa	Yes	20 – 21	Yes – incomplete	QDD & GHG and NTD & RIK	Partial
PcPG24	470 aa	No[b]		____		Partial
PcPG25	404 aa	Yes	22 – 23	Yes – incomplete	QDD & SHG and NTD & RIK	Full-length
PcPG26	189 aa	____				Partial
PcPG27	267 aa	____				Partial
PcPG28	185 aa	____				Partial
PcPG29	158 aa	____				Partial
PcPG30	158 aa	____				Partial
PcPG31	183 aa	____				Partial

[a]The sequence is missing nucleotides in the N-terminal region and has no signal peptide.

Table S6. Full-length and partial polygalacturonase sequences from *Phytophthora cinnamomi*

PG gene ID	Predicted protein (aa)	Signal peptide & position	GH28 domain	Catalytic (G/QDD & G/SHG) & Binding domains (NTD & RIK)	Full-length/ partial
PcPG32	175 aa	____			Partial
PcPG33	183 aa	____			Partial
PcPG34	138 aa	____			Partial
PcPG35	161 aa	____			Partial
PcPG36	73 aa	____			Partial
PcPG37	89 aa	____			Partial

[b]The sequence has an N-terminal region but no signal peptide.

[c]The sequence has no N-terminal region.

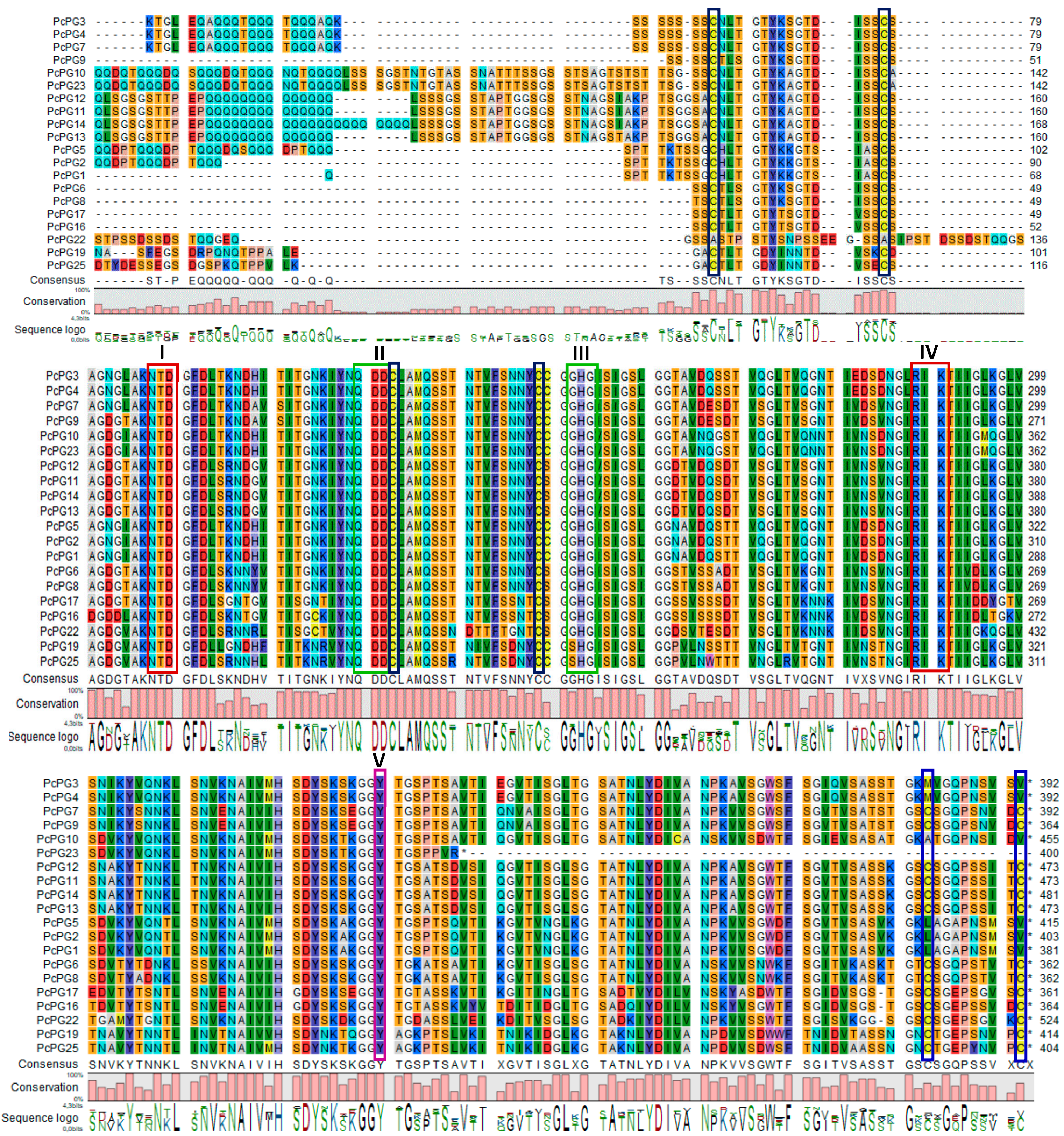

Figure S4. A multiple sequence alignment of the polygalacturonase protein sequences from *Phytophthora cinnamomi*. The sequences above contained the required length and signal peptide. The highly conserved cysteine regions (disulphide bridges) in the N-terminal region (top) middle region and C-terminal region (bottom), are marked with blue boxes as shown

above. The PGs analysed contained one to three disulphide bridges. All PG sequences contained the binding domains (NTD & RIK; Domain I & IV) marked in dark red boxes, and the catalytic domains (Q/KDD & G/SHG; Domain II & III) marked in green boxes. The invariantly conserved Y residue (Domain V) is marked by the purple box and present in all PGs analysed. PcPG23 lacked a region towards the end of the polypeptide chain, rendering the protein as a partial sequence.

Table S7: Log 2-Fold Change gene expression of PG genes from *P. cinnamomi* at 12 hpi, 24 hpi and 120 hpi during colonization of the susceptible avocado rootstock (R. 012) normalized to mycelia.

		Normalized read count in mycelia	12 hpi vs. mycelia		24 hpi vs. mycelia		120 hpi vs. mycelia	
**NCBI Gene ID**	**New Gene ID**	**Base Mean**	**Fold Change**	**padj**	**Fold Change**	**padj**	**Fold Change**	**padj**
OL334941	***pcpg1***	7,089050687	3,727133085	0,361369021	5,427063328	0,112128501	4,952595388	0,070323646
OL334945	***pcpg5***	12,53317935	7,179905644	0,011031321	5,68872165	0,047685514	9,253379578	2,17858E-06
OL334949	***pcpg9***	0,819837188	9,357377939	NA	8,82799846	NA	8,695524147	0,162009609
OL334956	***pcpg16***	92,69623888	-1,528584691	0,253232764	-1,667478691	0,116585993	-0,843269809	0,022920738
OL334957	***pcpg17***	0,164551001	5,576107637	NA	5,046727525	NA	2,367419336	NA
OL334959	***pcpg19***	4,816470763	0,622915905	NA	1,051579347	NA	-3,502069754	0,014409235
OL334962	***pcpg22***	19,22077692	0,785236322	0,781956919	4,683865954	0,000203064	-3,339728694	0,061227151
OL334965	***pcpg25***	0,972332323	9,357384537	NA	8,828005058	NA	8,933289208	0,149438017

References

1. Götesson A, Marshall JS, Jones DA, Hardham AR: **Characterization and evolutionary analysis of a large polygalacturonase gene family in the oomycete plant pathogen *Phytophthora cinnamomi***. *Molecular Plant-Microbe Interactions* 2002, **15**(9):907-921.
2. Liu H, Ma X, Yu H, Fang D, Li Y, Wang X, Wang W, Dong Y, Xiao B: **Genomes and virulence difference between two physiological races of *Phytophthora nicotianae***. *GigaScience* 2016, **5**(1):3.
3. Ali SS, Shao J, Lary DJ, Kronmiller BA, Shen D, Strem MD, Amoako-Attah I, Akrofi AY, Begoude BD, ten Hoopen GM: ***Phytophthora megakarya* and *Phytophthora palmivora*, closely related causal agents of cacao black pod rot, underwent increases in genome sizes and gene numbers by different mechanisms**. *Genome Biology and Evolution* 2017, **9**(3):536-557.
4. Tyler BM, Tripathy S, Zhang X, Dehal P, Jiang RH, Aerts A, Arredondo FD, Baxter L, Bensasson D, Beynon JL: ***Phytophthora* genome sequences uncover evolutionary origins and mechanisms of pathogenesis**. *Science* 2006, **313**(5791):1261-1266.
